# Photostability of polycyclic aromatic hydrocarbons in hydrated magnesium sulfate under Martian ultraviolet irradiation to assist organics detection on Mars

**DOI:** 10.1038/s41598-025-24253-8

**Published:** 2025-11-18

**Authors:** Andrew Alberini, Teresa Fornaro, Cristina García-Florentino, Giovanni Poggiali, Sole Biancalani, Francesco Renzi, Tommaso Grazioso, Nicola Tasinato, Daniela Alvarado Jiménez, Álvaro Vicente‐Retortillo, Maurizio Becucci, Edward A. Cloutis, Stephanie Connell, Giorgio Famiglini, Achille Cappiello, Germán M. Martinez, Mariano Battistuzzi, Christian Lorenz, Andrew Steele, Jesús Martínez-Frías, Felipe Gómez, Roger C. Wiens, Kevin P. Hand, John R. Brucato

**Affiliations:** 1 INAF- Astrophysical Observatory of Arcetri, L.go E. Fermi 5, Firenze, 50125 Italy; 2 Department of Physics and Astronomy, University of Florence, Sesto Fiorentino (FI), Via Giovanni Sansone 1, 50019 Sesto Fiorentino FI, Italy; 3 Department of Physics, University of Trento, Via Sommarive 14, 38123 Povo, Italy; 4 Italian Space Agency (ASI), viale del Politecnico snc, Rome, 00133 Italy; 5 Department of Earth Sciences, University of Florence, via G. La Pira 4, Florence, 50121 Italy; 6 University of Urbino Carlo Bo, Urbino (PU), Piazza Rinascimento 6, Urbino , 61029 Italy; 7 Scuola Normale Superiore, Pisa, Italy; 8 Centro de Astrobiología (CAB), CSIC-INTA, Torrejón de Ardoz, Spain; 9 Department of Chemistry, ‘Ugo Schiff’ - University of Florence, via della Lastruccia 3-13, Sesto Fiorentino (FI), 50019 Italy; 10 Centre for Terrestrial and Planetary Exploration, University of Winnipeg, Winnipeg, MB R3B 2E9 Canada; 11 Department of Earth, Atmospheric, and Planetary Sciences, Purdue University, Hampton Hall 550 Stadium Mall Drive, West Lafayette, IN 47907 USA; 12 Vancouver Island University, Nanaimo, BC Canada; 13 Lunar and Planetary Institute, Universities Space Research Association, Houston, TX USA; 14 Carnegie Institute for Science, Washington, DC USA; 15 Institute of Geosciences, IGEO (CSIC-UCM), Madrid, Spain; 16 Jet Propulsion Laboratory, California Institute of Technology, Pasadena, CA USA

**Keywords:** Polycyclic aromatic hydrocarbons, FTIR spectroscopy, UV irradiation, Mars 2020 perseverance rover mission, Planetary science, Astronomy and astrophysics

## Abstract

The Scanning Habitable Environments with Raman and Luminescence for Organics and Chemicals (SHERLOC) instrument, which is mounted on the Mars 2020 Perseverance rover, detected Raman signals in spectral regions relevant to organics, plausibly polycyclic aromatic hydrocarbons (PAHs), co-located with sulfate minerals, in the Quartier abrasion of the Issole outcrop in the Jezero crater floor on Mars. In order to ascertain the plausibility of the organic origin of these signals, it is essential to determine whether organics can withstand the effects of ambient Martian ultraviolet (UV) radiation after they are exposed by the Perseverance’s abrasion tool and prior to the analysis by SHERLOC. In this work, the stability under Martian-like UV irradiation of PAHs like 2,6-dihydroxynaphthalene and benzo[a]pyrene in hydrated magnesium sulfate, one of the main sulfate phases present in Quartier, is investigated. Our findings indicate that the spectroscopic features of 2,6-dihydroxynaphthalene and benzo[a]pyrene in hydrated magnesium sulfate remain unaltered when exposed to UV radiation comparable to that experienced at Jezero crater over a period of dozens of Martian days (or sols). Consequently, due to the photoprotective properties of this mineral, after the abrasion exposes them to the radiation, these compounds can still be detectable by the SHERLOC measurement and also by SuperCam because some organic bands fall in its infrared spectral range. In addition, photoproducts due to the UV exposure for both PAHs were detected. These results corroborate the hypothesis that the Raman signals detected by SHERLOC co-located with sulfates may arise from organic compounds.

**Supplementary Information:**

The online version contains supplementary material available at 10.1038/s41598-025-24253-8.

 Previous and ongoing Mars exploration missions have already confirmed that the planet was once habitable^1–3^. As a result, the search for biosignatures, such as the detection of organic compounds, has become a key objective of current Mars exploration programs^4^. Since then, additional organic compounds have been identified in Gale Crater by the Sample Analysis at Mars (SAM) suite aboard NASA’s Mars Science Laboratory (MSL) Curiosity rover^5–7^. However, because SAM measurements may alter the original Martian organic molecules, either through thermal degradation or chemical reactions^8,9^, non-destructive analytical techniques are essential. These methods can support the identification of pristine compounds, guide the selection of the most promising samples for further investigation and complement the use of more sensitive yet destructive techniques. NASA Mars 2020 Perseverance rover mission is operating now in the Jezero crater on Mars^10^ with the aim of detecting organic compounds in geological material. It is equipped with non-destructive instruments^10^ designed to detect organic compounds in geological materials and to collect samples that are astrobiologically relevant, with the ultimate goal of bringing them back to Earth via the Mars Sample Return (MSR) mission.

Although Perseverance relies on non-destructive techniques to detect organic compounds, it must still contend with the harsh Martian environment that threatens their preservation. Due to the planet’s extremely thin atmosphere (~ 6 mbar) composed predominantly of carbon dioxide (96%) and the lack of a global magnetic field, the surface is directly exposed to several degradation agents, including ultraviolet (UV) photons, Solar Energetic Particles (SEPs), and Galactic Cosmic Rays (GCRs), all of which can trigger the formation of reactive oxidants^2,11–14^.

Although UV photons penetrate only a few micrometers into the surface^15^, they remain particularly effective in degrading organic matter, especially in the mobile aeolian layer or in freshly abraded rock surfaces exposed by the rover.

Many organic compounds absorb UV light, leading to photochemical reactions and rapid UV-induced degradation over a period ranging from sols to a few years, depending on the mineral matrix hosting the organics^2^. This degradation occurs much faster than the effects caused by GCRs and SEPs, which unfold over hundreds of millions of years^16–18^.

Additionally, UV radiation can induce the photochemical formation of strong oxidizing agents, such as perchlorates, already detected in the Martian regolith^19^, which are capable of penetrating deep into the soil and potentially leading to the oxidative degradation of organic compounds present^20^.

When Perseverance performs an abrasion, for operational reasons, these exposed areas remain subject to environmental UV radiation for at least one sol before Perseverance’s proximity scientific instruments can take measurements. Therefore, any organic material present in the abraded areas must be stable under UV radiation for at least one sol in order to be detected by Perseverance’s proximity scientific instruments.

One of the Perseverance’s proximity science instruments, the Scanning Habitable Environments with Raman and Luminescence for Organics and Chemicals (SHERLOC)^21^, a deep UV Raman and fluorescence spectrometer, found interesting Raman features in spectral regions relevant to organics, co-located with sulfate minerals, in the Quartier abrasion of the Issole outcrop in the Jezero crater floor^22^. Interestingly, these features remained unaltered for 11 sols, from sol 293, when they were first detected, to sol 304, when their detection was reconfirmed^22^. Comparison with laboratory data indicate that these features are due to polycyclic aromatic hydrocarbons (PAHs)^23^. This would hold true only if those organic compounds in sulfates can withstand UV exposure for at least 11 sols.

Beyond the Quartier abrasion, Perseverance found intriguing targets during the exploration of Neretva Vallis, an ancient river channel that served as the primary inlet on Jezero crater’s western margin, across three rocks at Bright Angel zone^24^. Raman features in spectral regions relevant to organics were detected by SHERLOC in Apollo Temple abrasion (associated with carbonate and sulfate mineral phases), Walhalla Glades abrasion and Cheyava Falls rock surface. The Bright Angel rocks stand out as being particularly rich in organic matter compared to other targets analyzed by SHERLOC in Jezero Crater^24^. They represent a mudstone located outside Gale Crater that has been found to contain substantial amounts of organic carbon^25^, supporting the idea that organic compounds may have been widespread and accessible in ancient ($\:\sim\:3.5$ billion years old) Martian lakes and river systems.

The presence of sulfur and therefore sulfate minerals has always been a central focus of attention in the scientific community^26–28^. The transfer of sulfur, from eruption and initial emplacement, to aqueous and sedimentary processes, is one of the most important facets of Martian geochemistry, with its strong implications on climate and habitability at various stages in Mars history^26^. The presence of sulfur-rich compositions on Mars is suggested by meteorite data, in situ chemical and mineralogical analyses, remote sensing data from dust and surfaces, and geochemical models. Regarding the organic preservation, on Earth, sulfate minerals have shown to be able to preserve them^29–31^. On Mars, sulfate minerals might play a similar role, protecting organic compounds from the planet’s oxidizing surface conditions when trapped within intracrystalline inclusions^32,33^ or during post-depositional alterations, such as sulfate mineral growth from saline groundwater or neomorphic processes like recrystallization—a process well-documented in Mars’ history^34^. The extremely slow rates of sulfate ($\:{SO}_{4}^{2-}$) reduction would further support the preservation of trapped organics over extensive geological periods^33,35^. Additionally, studies by dos Santos et al.^36^ and by Alberini et al.^37^ demonstrated that sulfates can protect organics like amino acids and carboxylic acids from UV-induced damage, likely due to their opacity to UV radiation^23^. These findings suggest that sulfate-rich Martian sediments and rocks could be promising targets for the detection of preserved organic compounds^31,38^. However, it is no possible to generalize the sulfate mineral photoprotection because it depends on the nature of organics and their interaction with the mineral matrix^2^.

This study builds upon the methodological framework presented in Alberini et al.^37^, which investigated the photostability of aromatic carboxylic acids embedded in hydrated magnesium sulfate under Martian-like UV irradiation. Here, we extend this approach to PAHs, given their relevance to the in-situ Mars 2020 mission observations^23^, adsorbed in hydrated magnesium sulfate, which is one of the main sulfates detected in association with Quartier’s features of interest^39^. With this aim, spectroscopic and advanced mass spectrometry techniques have been used to assess UV-driven degradation pathways in order to inspect if the PAHs are plausible organic candidates for the Quartier abrasion as suggested by Fornaro et al.^23^. Specifically, we evaluated the photostability of the PAHs 2,6-dihydroxynaphthalene and benzo[a]pyrene within hydrated magnesium sulfate when subjected to Martian-like UV radiation to determine their likelihood of surviving at least $\:11\:sol$ of UV irradiation exposure.

Beyond Mars 2020 mission, investigating sulfates and their properties may also be useful for other Mars exploration missions, such as NASA’s Curiosity rover^40,41^, ESA’s upcoming ExoMars/Rosalind Franklin mission^42^ and orbital remote sensing campaigns. In particular, Curiosity is preparing to explore the layered rocks of Mount Sharp, where orbital data suggest the presence of sulfate minerals, potentially rich in magnesium, in various states of hydration. Furthermore, since sulfates are also widespread on other rocky bodies in the Solar System, such as the icy moons of the gas giants (e.g., Europa)^43,44^, and the volcanically active moon Io which is known to be enriched in sulfur^43^, the usefulness can also be extended to the upcoming Europa Clipper mission^45^ and the interpretation of spectroscopic data acquired by the MIRI instrument^46^ aboard the James Webb Space Telescope (JWST).

PAHs account for approximately $\:10\%$ of the total cosmic carbon^47^ and are responsible for infrared emissions ($\:3-15\:\mu\:m,\:3300-670\:{cm}^{-1}$) detected across the observable universe^48^. These compounds are produced in circumstellar regions through combustion processes and undergo transformation in the interstellar medium (ISM) due to exposure to UV and Lyman-α radiation, as well as shock waves^49,50^. After being integrated into icy or rocky bodies like asteroids and comets, they follow a different evolutionary path, including hydrothermal alterations within asteroids^51^ and radiation-induced changes in cometary ice^52^. The current estimated global carbon flux to Mars from cometary impacts is $\:13$ tons per year, and from asteroids $\:50$ tons per year^53,54^. The amount of organic material falling to the surface of Mars from Interplanetary Dust Particles (IDPs) and (micro)meteorites is estimated to be of the order of $\:1000$ tons per year^53,55^ and would have been higher in the past. The photochemical evolution of PAHs upon reaching planetary environments, including Mars and Earth, is of great interest, especially concerning the origin of life. It was estimated that $\:75\:\%$ of extraterrestrial organic matter in meteorites is aromatic^56^. These aromatic compounds, including PAHs, are more likely to survive atmospheric entry, as smaller molecules are often destroyed during the journey^57^. PAHs could later be broken down into smaller, biologically relevant molecules by photochemical catalysis in Martian ultraviolet radiation regimes^58^. Specifically, the presence of 2,6-dihydroxynaphthalene on Mars could be explained by both an exogenic and endogenic production. For the exogenic way, it could be a product of the H_2_O-naphthalene ice UV irradiation during the journey embedded in the interstellar dust grains and meteorites, resulting in multiple oxidations of the pristine naphthalene^59–61^. The presence of naphthalene in these kinds of environments is well literature-documented thanks to its detection in Cold Bokkeveld, Orgeuil, Asuka 881,458, Winchcombe and Murchison meteorites^56,62–65^. On the other hand, 2,6-dihydroxynaphthalene might have been also produced endogenously by hydroxylation of naphthalene during the wet-phase of Mars due to the presence of H_2_O-naphthalene solution and ionizing radiation^66,67^. Regarding the benzo[a]pyrene molecule, it was found in Cold Bokkeveld, Orgeuil, Asuka 881,458 and Murchison meteorites^56,62,64^.

Martian analog samples were prepared to simulate a possible natural interaction that might have occurred in an aqueous environment on early Mars between magnesium sulfate and the two PAHs 2,6-dihydroxynaphthalene and benzo[a]pyrene, followed by a desiccation event. The Martian analog samples were characterized by InfraRed (IR) from $\:8000\:{cm}^{-1}$ to $\:400\:{cm}^{-1}\:(1.25\:\mu\:m-25\:\mu\:m)$ in order to get insights into the possible molecule-mineral interactions, and compare with data acquired by another instrument on-board Perseverance, namely SuperCam^68^, which includes Visible and InfraRed (VISIR) reflectance spectroscopy (spectral range $\:400\:nm\:-\:900\:nm$, $\:1.3\:\mu\:m-\:2.6\:\mu\:m$).

Finally, the samples were irradiated with UV light in situ, and the degradation kinetics were measured using infrared spectroscopy. This approach enabled the assessment of the photostability of the PAHs in their pure state and when adsorbed on magnesium sulfate. The results obtained from this study offer valuable insights into the potential photoprotective or photocatalytic behavior of magnesium sulfate.

## Results and discussion

The band assignments for the pure 2,6-dihydroxynaphthalene and benzo[a]pyrene spectra were derived from both experimental and theoretical data available in the literature, as well as from the theoretical anharmonic calculations conducted in this study, especially concerning the assignment of combination and overtone bands. In general, there was a strong agreement between the observed band positions and those reported in the literature and theoretically calculated. As expected, some discrepancies arose due to differences in the physical state of the samples and/or variations in sample preparation methods and spectral acquisition techniques. In agreement with previous studies^20,37,69^, when molecules are adsorbed onto the mineral surface, a reduction in both the number and intensity of molecular bands compared to the pure organic compound was observed.

### 2,6-dihydroxynaphthalene IR characterization: band assignment and spectral changes due to molecular adsorption

**Fig. 1:**
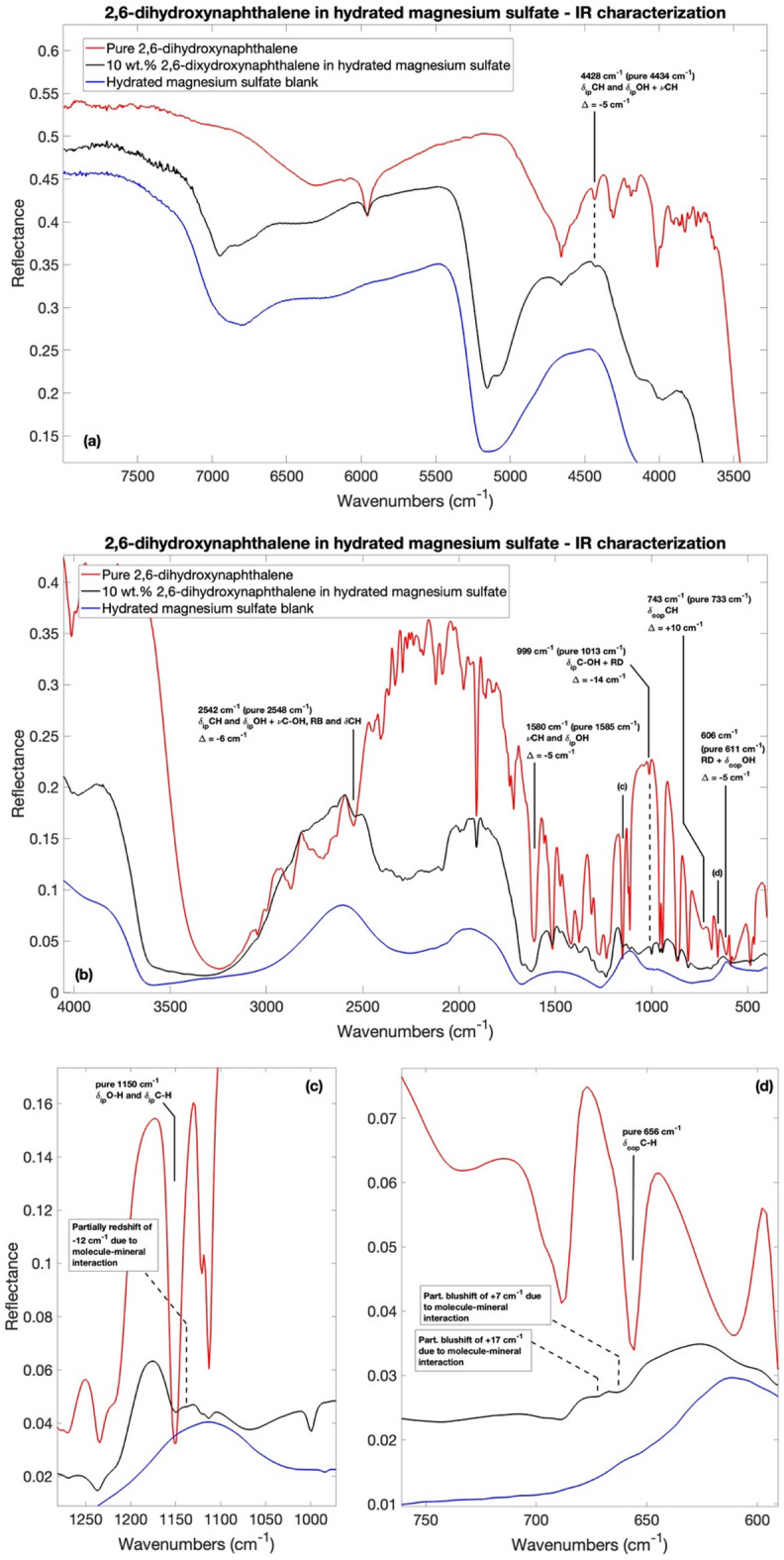
IR spectra comparison for pure 2,6-dihydroxynaphthalene (red spectrum), $\:10\:$wt.$\:\%$ 2,6-dihydroxynaphthalene adsorbed on hydrated magnesium sulfate (black spectrum) and hydrated magnesium sulfate blank (blue spectrum) in (**a**) IR $\:8000-3500\:{cm}^{-1}$ spectral range; (**b**) IR $\:3500-450\:{cm}^{-1}$ spectral range; (**c**) and (**d**) spectral zooms on 2,6-dihydroxynaphthalene partially shifted bands plausible due to molecule-mineral interaction. Only adsorbed 2,6-dihydroxynaphthalene bands with shifts greater than the resolution of the instrument ($\:>4\:{cm}^{-1}$) with respect to the pure 2,6-dihydroxynaphthalene are shown. Legend: $\:\nu\:$ stretching vibrations; $\:{\delta\:}_{ip}$ in-plane bending vibrations; $\:{\delta\:}_{oop}$ out-of-plane bending vibrations.

To demonstrate the differences in spectral characteristics before and after adsorption, Fig. 1 presents the IR spectra of pure 2,6-dihydroxynaphthalene, $\:10\:$wt.$\:\%$ 2,6-dihydroxynaphthalene adsorbed on hydrated magnesium sulfate, and the hydrated magnesium sulfate blank. As shown, several vibrational modes present in pure 2,6-dihydroxynaphthalene can no longer be clearly recognized when 2,6-dihydroxynaphthalene is adsorbed onto the mineral surface, or the corresponding bands appear significantly attenuated or hidden, likely due to matrix effects and changes in the local bonding environment. Interestingly, the spectral region displaying the most intense molecular bands after adsorption lies between $\:4700\:-\:3700$ $\:{cm}^{-1}\:(2.1-\:2.7\:\mu\:m)$, $\:2100\:-\:1800$ $\:{cm}^{-1}\:(4.8-5.6\:{\upmu\:}\text{m})$, and $\:1600\:-\:700$ $\:{cm}^{-1}$ $\:(6.3\:\ -14.3\:\mu\:m)$ (Fig. 1(a-d)). Beyond these ranges, significant absorptions related to hydrated magnesium sulfate are observed, corresponding to the vibrational modes of $\:{SO}_{4}$ and water in the mineral structure. Supplementary Table S1 provides a detailed list of all detectable bands in both the pure 2,6-dihydroxynaphthalene spectrum and 2,6-dihydroxynaphthalene adsorbed on hydrated magnesium sulfate, along with their respective band assignments and intensities.

As it can be appreciated in Fig. 1(a-d) and Supplementary Table S1, some shifts of molecular vibrational modes are present when the molecules are adsorbed on magnesium sulfate with respect to the pure molecule, specifically at $\:4434\:{cm}^{-1}$ ($\:2.26\:\mu\:m)$, $\:2548\:{cm}^{-1}$ $\:(3.92\:\mu\:m$), $\:1585$ $\:{cm}^{-1}\:(6.31\:\mu\:m$), $\:1150$ $\:{cm}^{-1}\:(8.70\:\mu\:m$), $\:1013$ $\:{cm}^{-1}\:(9.87\:\mu\:m$), $\:733$ $\:{cm}^{-1}\:(13.6\:\mu\:m$), $\:656$ $\:{cm}^{-1}\:(15.24\:\mu\:m$) and $\:611$ $\:{cm}^{-1}\:(16.4\:\mu\:m$), which are related mainly to OH and ring CH vibrations (Fig. 1(a-d)). In particular, the fundamental in-plane bending _ip_CH and _ip_OH band at $\:1150$ $\:{cm}^{-1}$ is partially shifted by $\:12$ $\:{cm}^{-1}$ to lower wavenumbers (Fig. 1(c)), meanwhile the fundamental out-of-plane bending _oop_CH band at $\:656$ $\:{cm}^{-1}$ is split and shifted by $\:7$ and $\:17$ $\:{cm}^{-1}$ to higher wavenumbers (Fig. 1(d)). A qualitative correlation between the two vibrational modes CH/OH and the direction of the shift is suggested: the bands shifted to lower wavenumbers are that with the OH contribution, meanwhile the only two bands with higher wavenumbers shift are only CH assigned, in particular to the out-of-plane CH bendings. For the negative OH-related shifts, this may indicate hydrogen bonds or weak interactions with the substrate, such as those between the OH groups of the molecule and the sulfate groups ($\:{SO}_{4}^{2-}$) or the water molecules in hydrated magnesium sulfate structure. However, the rest of the bands of 2,6-dihydroxynaphthalene do not present any shifts or the shift intensity are under the $\:4$ $\:{cm}^{-1}$ interferometer resolution.

Alterations caused by possible interactions with the mineral could also modify the sulfate S-O bond length, and thus the symmetry of the anions, which would cause the bands associated with the mineral to shift in the IR spectrum. However, the characteristic spectral region of sulfate stretching and bending absorption, $\:1000-1250$ $\:{cm}^{-1}$ ($\:8-10\:\mu\:m$)^70^, is mostly covered by 2,6-dihydroxynaphthalene absorption bands. The band expected between $\:615$ $\:{cm}^{-1}$ and $\:620$ $\:{cm}^{-1}$ ($\:16.3$ µm and $\:16.1$ µm) due to the bending mode of the sulfate^71–73,75^ is covered even in the epsomite blank by the broad water related libration band at a bit higher wavenumber around $\:750$ $\:{cm}^{-1}$ ($\:13.3\:\mu\:m$). Therefore, it is not possible to evaluate any interactions between the organic and the mineral by analyzing the sulfate IR bands.

Finally, there is evidence of spectral changes in the spectrum of 2,6-dihydroxynaphthalene adsorbed on hydrated magnesium sulfate with respect to the mineral blank regarding the water-associated mineral bands. To better understand the hydration state of our samples, we measured IR spectra of kieserite (MgSO_4_$\:\cdot\:$H_2_O) and hexahydrite (MgSO_4_$\:\dot\:6$H_2_O) (Supplementary Figure S1) to compare with the spectrum of the mineral blank and the mineral after the organic adsorption. In addition, a comparison with the data from Bonello et al. work^76^ showing the spectra of magnesium sulfate with different hydration states was done. The $\:3583$ $\:{cm}^{-1}$ ($\:2.8\:\mu\:m$) OH stretching band^75^ undergoes a change in the overall shape of the band, consisting in a narrowing and the observation of a feature likely due to the contribution of the OH-band belonging to 2,6-dhn at lower wavenumbers. The $\:5161$ $\:{cm}^{-1}$ ($\:1.9\:\mu\:m$) combination of water OH stretching and OH bending^77^ band shape changes with a narrowing and the arising of two distinct peaks. Finally, the $\:6812$ $\:{cm}^{-1}$ ($\:1.5\:\mu\:m$) $\:1$^st^ overtone of the water OH stretching^77^ undergoes a similar fate of the previous one showing two distinct peaks after the adsorption of the 2,6-dhn with a shift to higher wavenumber of $\:135$ $\:{cm}^{-1}$ ($\:6947$ $\:{cm}^{-1}$) in this case.

Since the shape and position of the water-associated bands reflect the hydration level of magnesium sulfate in the IR spectra^76^ (Supplementary Figure S1), we are confident that the sample did not undergo sufficient dehydration to result in kieserite formation. In fact, the kieserite water-associated bands are non-consistent with the ones of the mineral blank and the organics adsorbed onto the mineral spectra^76^ (Supplementary Figure S1). A possible explanation of these features could be multiple magnesium sulfate hydration states, to higher hydration level than kieserite, that could co-exist in the sample.

### Benzo[a]pyrene IR characterization: band assignment and spectral changes due to molecular adsorption

**Fig. 2:**
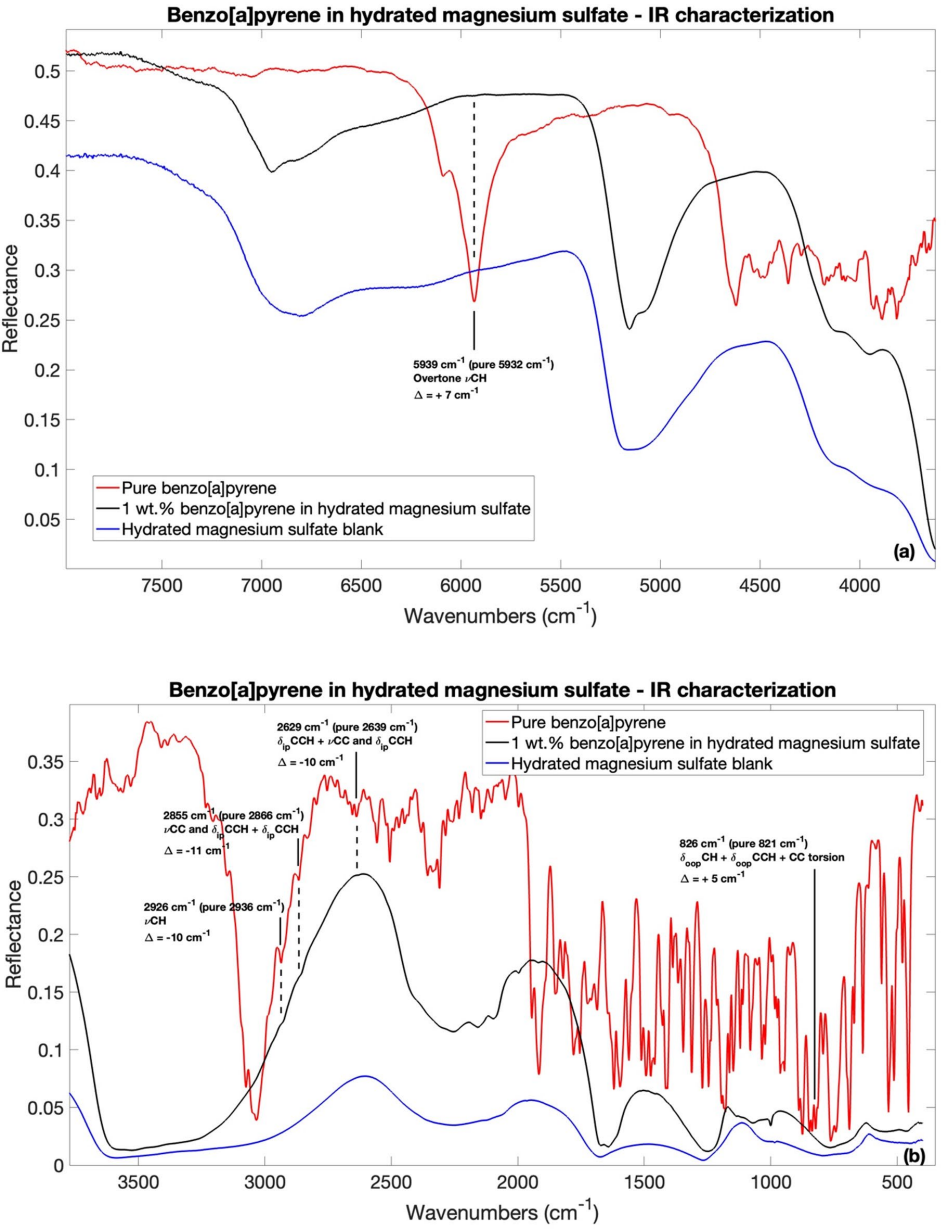
IR spectra comparison for pure benzo[a]pyrene (red spectrum), $\:1\:$wt.$\:\%$ benzo[a]pyrene adsorbed on hydrated magnesium sulfate (black spectrum) and hydrated magnesium sulfate blank (blue spectrum) in (**a**) IR $\:8000-3500\:{cm}^{-1}$ spectral range; (**b**) IR $\:3500-450\:{cm}^{-1}$ spectral range. Only adsorbed benzo[a]pyrene bands with shifts greater than the resolution of the instrument ($\:>4\:{cm}^{-1}$) with respect to the pure benzo[a]pyrene are shown. Legend: $\:\nu\:$ stretching vibrations; $\:{\delta\:}_{ip}$ in-plane bending vibrations; $\:{\delta\:}_{oop}$ out-of-plane bending vibrations.

To demonstrate the differences in spectral characteristics before and after adsorption, Fig. 2 presents the IR spectra of pure benzo[a]pyrene, $\:1$ wt.$\:\%$ benzo[a]pyrene adsorbed on hydrated magnesium sulfate, and the hydrated magnesium sulfate blank. As shown, several vibrational modes characteristic of pure benzo[a]pyrene are either no longer recognizable or significantly attenuated when the molecule is adsorbed onto the mineral surface. This behavior may result from a combination of factors, including the dilution of the organic compound within the mineral matrix ($\:1$ wt%), which can hamper its spectral detectability, as well as potential matrix effects, changes in the local bonding environment, and alterations in vibrational selection rules due to interactions with sulfates^78^. Interestingly, the spectral region displaying the most intense molecular bands after adsorption lies between $\:6000\:-\:4500$ $\:{cm}^{-1}\:(1.7-2.2\:\mu\:m)$, $\:2700\:-\:2500$ $\:{cm}^{-1}\:(3.7-4.0\:{\upmu\:}\text{m})$, and $\:2000\:-\:1400$ $\:{cm}^{-1}$ $\:(5.0\: -7.1\:\mu\:m)$ (Fig. 2(a) and Fig. 2(b)). Beyond these ranges, significant absorptions related to hydrated magnesium sulfate are observed, corresponding to the vibrational modes of $\:{SO}_{4}$ and water in the mineral structure. Supplementary Table S2 provides a detailed list of all detectable bands in both the pure benzo[a]pyrene spectrum and benzo[a]pyrene adsorbed on hydrated magnesium sulfate, along with their respective band assignments and intensities.

Some shifts of molecular vibrational modes are present when the molecules are adsorbed on magnesium sulfate with respect to the pure molecule, specifically at $\:5932\:{cm}^{-1}$ ($\:1.7\:\mu\:m)$, $\:2936\:{cm}^{-1}$ ($\:3.4\:\mu\:m$), $\:2866\:{cm}^{-1}$ ($\:3.5\:\mu\:m$), and $\:2639\:{cm}^{-1}$ $\:(3.8\:\mu\:m$) and $\:821\:{cm}^{-1}$ $\:(12.2\:\mu\:m$), which are mainly assigned to CCH vibrations (Fig. 2(a) and Fig. 2(b)). The rest of the bands of benzo[a]pyrene do not present any shifts or the shift intensity are under the $\:4$ $\:{cm}^{-1}$ interferometer resolution. The $\:5932\:{cm}^{-1}$ (Overtone CH vibrations) and $\:821\:{cm}^{-1}$ (CCH and CCC vibrations) bands, have a positive shift of $\:{+7\:cm}^{-1}$ and $\:+5$ $\:{cm}^{-1}$ respectively, meanwhile the $\:2936\:{cm}^{-1}$, $\:2866\:{cm}^{-1}$ and $\:2639\:{cm}^{-1}$ bands, related mainly to CCH vibrations, have negative shifts of $\:{-10\:cm}^{-1},\:{-11\:cm}^{-1}$ and $\:{-10\:cm}^{-1}$, respectively. These molecular band shifts suggest a benzo[a]pyrene-mineral interaction.

As for the 2,6-dhn case study, there is evidence of spectral changes in the spectrum of benzo[a]pyrene adsorbed on hydrated magnesium sulfate with respect to the mineral blank regarding the water-associated mineral bands. Also in this case, to better understand the hydration state of our samples, together with Bonello et al.^77^ reference spectra, we reported the IR spectra of kieserite (MgSO_4_$\:\cdot\:$H_2_O) and hexahydrite (MgSO_4_$\:\cdot\:6$H_2_O) (Supplementary Figure S2) to compare with the spectrum of the mineral blank and the mineral after the organic adsorption.

The $\:3583$ $\:{cm}^{-1}$ ($\:2.8\:\mu\:m$) OH stretching band^75^ band undergo a change in the overall shape of the band, consisting in a narrowing and a shift to lower wavenumber of $\:55\:{cm}^{-1}$ ($\:3528$ $\:{cm}^{-1}$).

The $\:5161$ $\:{cm}^{-1}$ ($\:1.9\:\mu\:m$) combination of water OH stretching and OH bending^77^ band changes in two distinct peaks and a narrowing of the band.

Finally, the $\:6812$ $\:{cm}^{-1}$ ($\:1.5\:\mu\:m$) $\:1$^st^ overtone of the water OH stretching^77^ undergo a similar fate of the previous one showing two distinct peaks after the adsorption of the 2,6-dhn with this time a shift to higher wavenumber of $\:135$ $\:{cm}^{-1}$ ($\:6949$ $\:{cm}^{-1}$).

As for the 2,6-dhn case study, we are confident that dehydration of the benzo[a]pyrene sample leading to kieserite formation can be excluded. This conclusion is supported by the fact that the water-associated vibrational bands of kieserite are inconsistent with those observed in both the mineral blank and the spectra of the organic compound adsorbed on the mineral^76^ (Supplementary Figure S2). Altogether, these observations suggest that the detected features are more likely attributable to the coexistence of multiple hydration states of magnesium sulfate in the sample, with hydration levels higher than that of kieserite.

### UV irradiation of 2,6-dihydroxynaphthalene and $\:10\:$wt% 2,6-dihydroxynaphthalene on hydrated magnesium sulfate

Table 1 reports the bands analyzed for the study of the photodegradation kinetics of pure 2,6-dihydroxynaphthalene (2,6-dhn) with the corresponding assignments based on DFT calculations carried out in this work and literature^79^, degradation rate ( $\:\beta\:$ ), half-lives ( $\:{t}_{1/2}\:$**)**, destruction cross section ( $\:{\upsigma\:}$ ) and degradation percentage ( DP% ) results from the fit model described in the Methods section.

**Table 1:** Degradation rate ( $\:\varvec{\beta\:}$ ), half-lives ( $\:{\varvec{t}}_{1/2}\:$), destruction cross section ( $\:\varvec{\upsigma\:}$ ) and degradation percentage for pure 2, 6-dhn, along with vibrational mode assignment (See Fig. 3 caption for the legend). Half-life values are evaluated both relative to the annual mean UV flux at Jezero crater (estimated using the COMIMART model^80^ which includes state-of-the-art dust radiative properties^81,82^ and corrected by Mastcam-Z opacities^37,83^) and relative to the theoretical Martian UV flux estimated by patel et al. ^12^ (assuming dust free atmosphere at the noontime equator^4^). The last row weighting procedure is showed in supplementary table S3.

Band [$\:{\varvec{c}\varvec{m}}^{-1}$]	2,6-dihydroxynaphthalene vibrational mode	$\:\varvec{\beta\,}\left[{{10}^{-4}\varvec{s}}^{-1}\right]$	$\:{\varvec{t}}_{1/2}\:\left[\varvec{s}\varvec{o}\varvec{l}\right]$ Jezero crater flux	$\:{\varvec{t}}_{1/2}\:\left[\varvec{s}\varvec{o}\varvec{l}\right]$ Patel et al., 2002 flux	$\:\varvec{\sigma\,}//\left[{10}^{-21}{\varvec{c}\varvec{m}}^{2}\right]$	Degradation percentage DP [%]
4326	Combination δ_ip_CH and δ_ip_OH + 𝜈CH *	$\:6\pm\:3$	8 ± 3	$\:5\pm\:2$	$\:2.4\pm\:1.0$	$\:1.2\pm\:0.2$
2899	Combination δ_ip_CH and δ_ip_OH and 𝜈CC + δ_ip_CH and 𝜈CC and δOH *	$\:1.5\pm\:1.2$	36 ± 26	$\:21\pm\:17$	$\:0.5\pm\:0.4$	$\:2.3\pm\:1.3$
2874	Fundamental 𝜈CH *^/^**	$\:2.2\pm\:0.5$	24 ± 6	$\:14\pm\:3$	$\:0.8\pm\:0.2$	$\:1.0\pm\:0.1$
2706	Combination δ_ip_CH and δ_ip_OH + δ_ip_CH and 𝜈CC (adjacent to OH) and δ_ip_OH *	$\:1.3\pm\:0.4$	40 ± 11	$\:23\pm\:7$	$\:0.5\pm\:0.1$	$\:1.5\pm\:0.3$
2687	Combination 𝜈C-OH and ring breathing and δCH + δ_ip_CH and δ_ip_OH and 𝜈CC *	$\:2.0\pm\:1.0$	27 ± 14	$\:16\pm\:8$	$\:0.7\pm\:0.4$	$\:2.0\pm\:0.5$
2548	Combination δ_ip_CH and δ_ip_OH + 𝜈C-OH and ring breathing and δCH *	$\:1.5\pm\:0.3$	36 ± 7	$\:21\pm\:4$	$\:0.5\pm\:0.1$	$\:0.8\pm\:0.1$
2448	Combination δ_ip_CH and δ_ip_OH + δ_ip_CH and δ_ip_OH *	$\:3.2\pm\:0.7$	18 ± 5	$\:10\pm\:2$	$\:1.1\pm\:0.3$	$\:1.3\pm\:0.1$
2133	Combination δ_oop_CH + δ_ip_CH and δ_ip_OH *	$\:1.9\pm\:0.9$	28 ± 13	$\:16\pm\:8$	$\:0.7\pm\:0.3$	$\:2.8\pm\:0.7$
2122	Combination δ_ip_CH and δ_ip_C-OH + 𝜈CC and δ_ip_OH and δ_ip_CH *	$\:1.9\pm\:0.8$	29 ± 13	$\:17\pm\:8$	$\:0.7\pm\:0.3$	$\:0.4\pm\:0.1$
2089	Combination δ_ip_CH and 𝜈CC and δOH + ring deformation *	$\:3.2\pm\:0.9$	17 ± 5	$\:10\pm\:3$	$\:1.1\pm\:0.3$	$\:0.4\pm\:0.1$
2071	Combination δ_ip_CH and 𝜈CC and δ_ip_OH + ring deformation *	$\:4.2\pm\:1.4$	13 ± 5	$\:7\pm\:3$	$\:1.5\pm\:0.5$	$\:1.4\pm\:0.2$
2006	Combination δ_oop_CH + δ_ip_CH *	$\:3.6\pm\:1.3$	15 ± 5	$\:8\pm\:3$	$\:1.3\pm\:0.5$	$\:2.6\pm\:0.3$
1312	Fundamental δ_ip_OH *^/^**	$\:11\pm\:2$	5 ± 1	$\:2.9\pm\:0.6$	$\:3.9\pm\:0.9$	$\:0.55\pm\:0.04$
1281	Fundamental δ_ip_CH *^/^**	$\:2.7\pm\:0.6$	20 ± 4	$\:11\pm\:3$	$\:1.0\pm\:0.2$	$\:0.8\pm\:0.1$
1236	Fundamental δ_ip_CH *^/^**	$\:5.7\pm\:1.4$	9 ± 2	$\:5.4\pm\:1.4$	$\:2.1\pm\:0.5$	$\:0.51\pm\:0.04$
1215	Fundamental δ_ip_OH *	$\:4.4\pm\:0.7$	12 ± 2	$\:7.0\pm\:1.1$	$\:1.6\pm\:0.3$	$\:1.2\pm\:0.1$
1150	Fundamental δ_ip_CH and δ_ip_OH *	$\:3.3\pm\:0.8$	16 ± 4	$\:9\pm\:2$	$\:1.2\pm\:0.3$	$\:0.31\pm\:0.03$
1065	Combination δ_oop_CH + δ_oop_CH *	$\:3.4\pm\:0.9$	16 ± 4	$\:9\pm\:2$	$\:1.2\pm\:0.3$	$\:30\pm\:3$
1040	Combination δ_oop_OH + δ_oop_CH *	$\:6\pm\:3$	10 ± 5	$\:6\pm\:3$	$\:2.0\pm\:1.0$	$\:2.2\pm\:0.4$
Weighted average values		$\:2.3\pm\:0.2$	$\:10\pm\:1$	$\:5.7\pm\:0.4$	$\:0.8\pm\:0.1$	$\:0.55\pm\:0.02$

* ^80^ ; ** DFT calculations (this work).

**Fig. 3:**
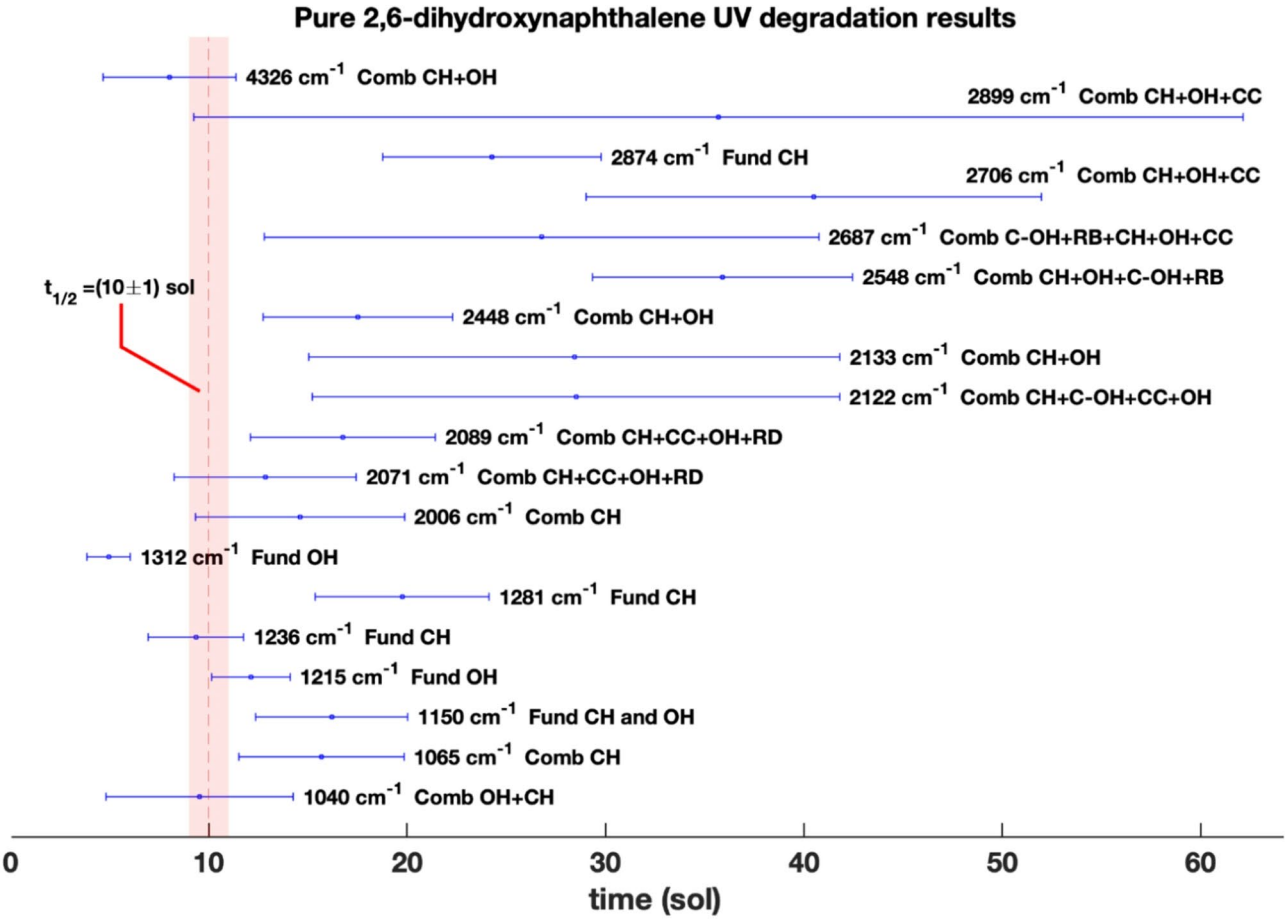
Pure 2,6-dihydroxynaphthalene half-lifetime degradation values$\:\:\left(sol\right)$ relative to the Jezero UV flux for the molecular bands mainly assigned to OH and ring CH groups. The vertical red dashed line is the weighted average half-lifetime. Legend: CC, CH and OH vibrations are carbon-carbon, carbon-hydrogen and oxygen-hydrogen vibrations, respectively; C-OH vibration involve the bond between the carbon atom and the OH functional group; RD and RB are the ring deformation and ring breathing vibrations and they affect all the molecular ring structure.

As observed in Table 1 and Fig. 3, nineteen molecular bands mainly assigned to OH and ring CH vibrations show a degradation in the pure molecule as a consequence of UV irradiation (See supplementary Figure S3). At the bottom of Table 1 weighted average values are reported.

In particular, bands with wavenumbers less than $\:\sim\:2100\:{cm}^{-1}$ show lower half-lifetimes, thus a faster degradation rate. These include all the analyzed fundamental molecular vibrational modes at $\:1312$ $\:{\:cm}^{-1}$ (Fundamental 𝛿_ip_OH), $\:1281$ $\:{\:cm}^{-1}$ (Fundamental 𝛿_ip_CH), $\:1236\:{\:cm}^{-1}$(Fundamental 𝛿_ip_CH), $\:1215\:{\:cm}^{-1}$ (Fundamental 𝛿_ip_OH), and $\:1150$ $\:{\:cm}^{-1}$ (𝛿_ip_CH and 𝛿_ip_OH), except the $\:2874$ $\:{cm}^{-1}$ band (Fundamental 𝜈CH). At wavenumbers above $\:2100{\:cm}^{-1}$, more combination bands come into play where vibrational modes are mixed and degradation times lengthen. The observation that some combination bands degrade more slowly may be due to the fact that they involve highly stable bonds, which contribute to their persistence.

Among the 2,6-dihydroxynaphthalene (2,6-dhn) bands analyzed, the one with the fastest degradation is the $\:1312$ $\:{\:cm}^{-1}$, assigned to the fundamental 𝛿_ip_OH vibration, with an half-lifetime of $\:{t}_{1/2}^{1312}=\left(5\pm\:1\right)\:sol$ and cross section $\:{{\upsigma\:}}_{1312}=\left(3.9\pm\:0.9\right)\:{10}^{-21}{cm}^{2}$ relative to the Jezero UV flux (Table 1). This is expected as the OH functional group is the most labile in the 2,6-dhn structure. Overall, the weighted mean half-lifetime value for all the 2,6-dhn bands analyzed is $\:{t}_{1/2}^{\overline{\text{2,6}-dhn}}=\left(10\pm\:1\right)\:sol$ with a cross section of $\:{{\upsigma\:}}^{\overline{\text{2,6}-dhn}}=\left(0.8\pm\:0.1\right)\cdot\:{10}^{-21}\:{cm}^{2}$ relative to the Jezero UV flux (Table 1). Noteworthy, the weighted average amount of molecule degraded at the end of the UV experiment, i.e. the degradation percentage (DP), is $\:{\text{D}\text{P}}^{\overline{\text{2,6}-dhn}}=\left(0.55\pm\:0.02\right)\:\%$. DP values are not constant along the IR spectrum: above $\:2100{\:cm}^{-1},\:$molecular bands are more degraded, even though their rate of degradation is slower as previously discussed.

On the other hand, the results for the 2,6-dhn adsorbed on hydrated magnesium sulfate are very different. The only adsorbed molecular bands that degrade when 2,6-dhn is adsorbed on hydrated magnesium sulfate are at $\:2444\:{cm}^{-1}$, $\:2135\:{cm}^{-1}$ and $\:2091\:{cm}^{-1}$, with half-lifetimes $\:{t}_{2444}^{adsorbed}=\left(18.8\pm\:1.5\right)\:sol$, $\:{t}_{2135}^{adsorbed}=\left(17\pm\:3\right)\:sol$ and $\:{t}_{2091}^{adsorbed}=\left(23\pm\:2\right)\:sol$, which are consistent with the corresponding half-lifetimes observed in the case of pure 2,6-dhn (See Supplementary Figure S4). For all the other molecular bands, no significant degradation is observed for the entire duration of our UV irradiation experiment, suggesting a photoprotective behavior of this mineral toward the 2,6-dhn. For some bands, signals of change in the adjacent continuum not associated with band degradation can be observed.

**Fig. 4:**
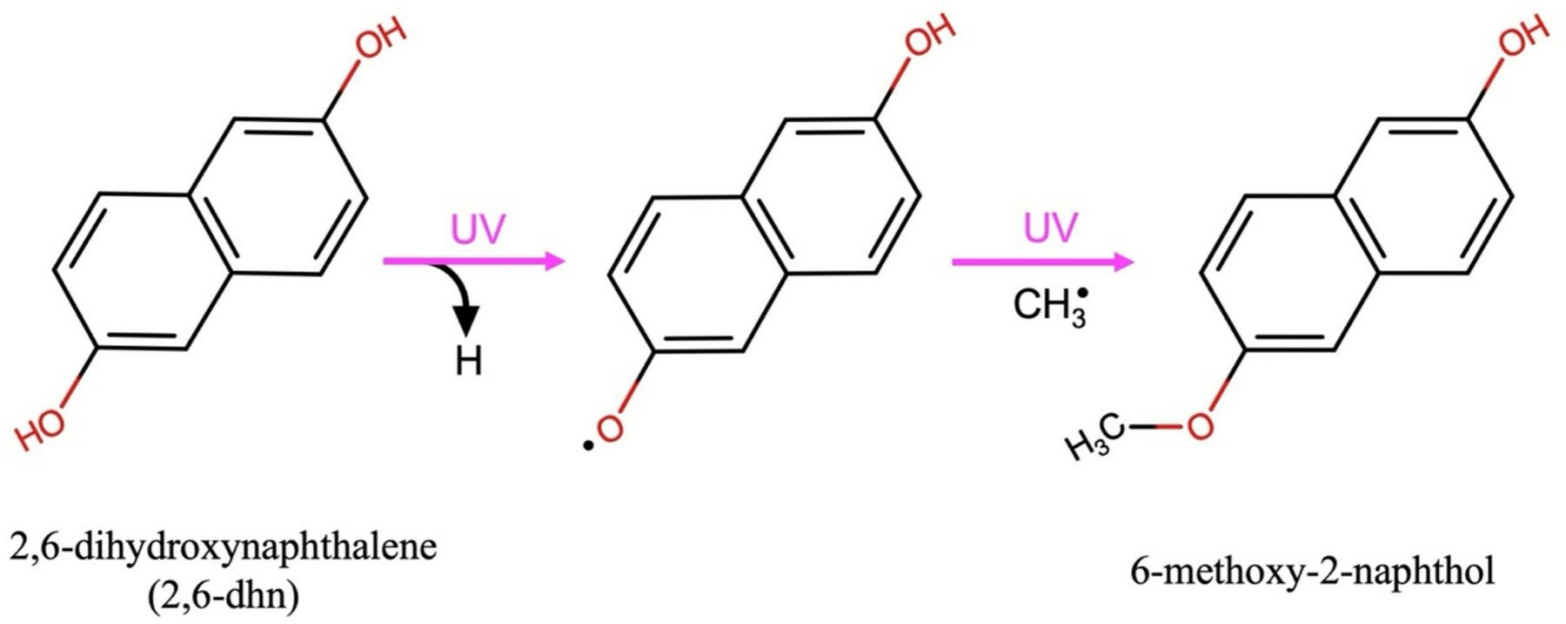
Suggested 6-methoxy-2-naphthol photoproduct formation from 2,6-dihydroxynaphthalene through radical processes due to the UV irradiation exposure.

Since IR spectroscopy has limited sensitivity for detecting photoproducts formed at low concentrations, the more sensitive Liquid Chromatography - Liquid Electron Ionization - High Resolution Mass Spectrometry (LC-LEI-HRMS) technique was employed. Using the latter technique, the irradiated pure 2,6-dhn sample revealed the formation of 6-methoxy-2-naphthol as a photoproduct, where a tentative radical chemical pathway is showed in Fig. 4.

A hypothesis for the formation of the 6-methoxy-2-naphthol could arrive from the fastest degradation of the OH bond of the initial molecule due to the methylation of the alcohol probably through the formation of radicals. As observed in the IR spectroscopy experiment (Fig. 3), in the initial molecule not only the OH bonds are degraded but also the CH and CC bonds at a longer irradiation time. This suggest that part of the initial molecules degrade even further probably leading to the formation of small organic molecule fragments (such as $\:{CH}_{3}^{.}$) that can react with a less degraded initial molecule where still only the OH bond is broken (Fig. 4). This photoproduct, 6-methoxy-2-naphthol, was not detected in the pre-irradiated sample of pure 2,6-dhn, confirming that it is generated as a result of the UV exposure. Conversely, when the same technique was applied to investigate the photoproduct formation in the irradiated and non-irradiated 10 wt% 2,6-dhn adsorbed on the hydrated magnesium sulfate, no photoproducts were detected. A possible explanation for the photoproduct absence could be found in the molecular OH functional group stabilization due to the interaction with the mineral, which makes the formation of the oxygen radical more difficult (Fig. 4). In fact, in the IR spectrum of the 2,6-dhn adsorbed on the magnesium sulfate, many of the bands that present a contribution of a vibrational mode assigned to the OH bond is shifted or splitted in two bands, indicating an interaction through this functional group.

### UV irradiation of benzo[a]pyrene and $\:1\:$wt% benzo[a]pyrene on hydrated magnesium sulfate

Table 2 reports the bands analyzed for the study of the photodegradation kinetics of pure benzo[a]pyrene with the corresponding assignments based on DFT calculations carried out in this work and literature^84^, degradation rate ( $\:\beta\:$ ), half-lives ( $\:{t}_{1/2}\:$**)**, destruction cross section ( $\:{\upsigma\:}$ ) and degradation percentage ( DP% ) results from the fit model described in the Methods section.

**Table 2:** Degradation rate ( $\:\varvec{\beta\:}$) , half-lives ( $\:{\varvec{t}}_{1/2}\:$), destruction cross section ( $\:\varvec{\upsigma\:}$ ) and degradation percentage for pure benzo[a]pyrene, along with vibrational mode assignment (See Fig. 5 caption for the legend). Half-life values are evaluated both relative to the annual mean UV flux at Jezero crater (estimated using the COMIMART model^80^ which includes state-of-the-art dust radiative properties^81,82^ and corrected by Mastcam-Z opacities^37,83^) and relative to the theoretical Martian UV flux estimated by patel et al. ^12^ (assuming dust free atmosphere at the noontime equator^4^). The last row weighting procedure is showed in supplementary table S3.

Band [$\:{\varvec{c}\varvec{m}}^{-1}$]	benzo[a]pyrene vibrational mode	$\:\varvec{\beta\:}\:\left[{{10}^{-3}\varvec{s}}^{-1}\right]$	$\:{\varvec{t}}_{1/2}\:\left[\varvec{s}\varvec{o}\varvec{l}\right]$ Jezero crater flux	$\:{\varvec{t}}_{1/2}\:\left[\varvec{s}\varvec{o}\varvec{l}\right]$ Patel et al., 2002 flux	$\:\varvec{\sigma\:}\:\left[{10}^{-20}{\varvec{c}\varvec{m}}^{2}\right]$	Degradation percentage [%]
5932	Overtone 𝜈CH **	$\:2.2\pm\:0.8$	2.4 ± 0.9	$\:1.4\pm\:0.5$	$\:0.8\pm\:0.3$	$\:0.36\pm\:0.04$
4621	Comb 𝜈CH and 𝜈CC + 𝛿_ip_CCH**	$\:1.8\pm\:0.5$	2.9 ± 0.7	$\:1.7\pm\:0.4$	$\:0.9\pm\:0.2$	$\:0.70\pm\:0.06$
1620	Comb 𝛿_ip_CCH + 𝛿_ip_CCC and 𝛿_ip_CCH *^/^**	$\:1.7\pm\:0.5$	3.2 ± 0.9	$\:1.9\pm\:0.5$	$\:0.6\pm\:0.2$	$\:5.1\pm\:0.5$
1597	Comb 𝜈CC + 𝛿_ip_CCH *^/^**	$\:1.3\pm\:0.5$	4.1 ± 1.5	$\:2.4\pm\:0.9$	$\:0.5\pm\:0.2$	$\:1.5\pm\:0.2$
1560	Comb 𝜈CC + 𝛿_ip_CCH *^/^**	$\:1.7\pm\:0.5$	3.2 ± 0.9	$\:1.8\pm\:0.5$	$\:0.6\pm\:0.2$	$\:2.4\pm\:0.2$
1512	Comb 𝛿_ip_CCH + 𝛿_ip_CCC *^/^**	$\:2.0\pm\:0.5$	2.7 ± 0.6	$\:1.6\pm\:0.4$	$\:0.7\pm\:0.2$	$\:1.9\pm\:0.1$
1493	Comb 𝜈CC + 𝛿_ip_CCH *^/^**	$\:1.7\pm\:0.5$	3.2 ± 0.9	$\:1.8\pm\:0.5$	$\:0.6\pm\:0.2$	$\:4.7\pm\:0.4$
1427	Comb 𝛿_oop_CH + 𝜏CC and 𝛿_oop_CH *^/^**	$\:1.7\pm\:0.5$	3.2 ± 0.9	$\:1.8\pm\:0.5$	$\:0.6\pm\:0.2$	$\:5.7\pm\:0.6$
1245	Comb 𝛿_ip_CCH + 𝜈CC *^/^**	$\:2.0\pm\:0.4$	2.7 ± 0.6	$\:1.6\pm\:0.4$	$\:0.7\pm\:0.2$	$\:4.0\pm\:0.3$
1213	Comb 𝛿_ip_CCH and 𝛿_ip_CCC + 𝛿_ip_CCH and 𝛿_ip_CCC **	$\:2.8\pm\:0.6$	1.9 ± 0.4	$\:1.1\pm\:0.2$	$\:1.0\pm\:0.2$	$\:13.3\pm\:0.9$
1194	Fund 𝛿_ip_CCH *^/^**	$\:3.6\pm\:0.6$	1.5 ± 0.3	$\:0.8\pm\:0.2$	$\:1.3\pm\:0.2$	$\:10.9\pm\:0.7$
1163	Fund 𝛿_ip_CCH *^/^**	$\:1.4\pm\:0.4$	3.7 ± 1.1	$\:2.1\pm\:0.6$	$\:0.5\pm\:0.2$	$\:2.7\pm\:0.3$
1121	Comb 𝛿_oop_CH and 𝜏CC + 𝛿_oop_CH and 𝜏CC *^/^**	$\:2.5\pm\:0.5$	2.1 ± 0.4	$\:1.2\pm\:0.3$	$\:0.9\pm\:0.2$	$\:4.0\pm\:0.3$
1035	Comb 𝛿_ip_CCC + 𝛿_ip_CCC and 𝛿_ip_CCH *^/^**	$\:3.0\pm\:0.6$	1.7 ± 0.3	$\:1.0\pm\:0.2$	$\:1.1\pm\:0.2$	$\:4.8\pm\:0.3$
1021	Comb 𝜈CC + 𝛿_ip_CCH *^/^**	$\:3.3\pm\:0.6$	1.6 ± 0.3	$\:0.9\pm\:0.2$	$\:1.2\pm\:0.2$	$\:10.9\pm\:0.7$
974	Fund 𝛿_oop_CH *^/^**	$\:2.5\pm\:0.5$	2.1 ± 0.4	$\:1.2\pm\:0.3$	$\:0.9\pm\:0.2$	$\:11.8\pm\:0.8$
962	Fund 𝛿_oop_CH *^/^**	$\:2.8\pm\:0.5$	1.9 ± 0.4	$\:1.1\pm\:0.2$	$\:1.0\pm\:0.2$	$\:4.5\pm\:0.3$
889	Comb 𝛿_ip_CCC + 𝛿_ip_CCH *	$\:3.7\pm\:0.6$	1.4 ± 0.2	$\:0.8\pm\:0.1$	$\:1.3\pm\:0.2$	$\:5.0\pm\:0.3$
847	Comb 𝛿_oop_CH + 𝜏CC **	$\:3.6\pm\:0.6$	1.5 ± 0.3	$\:0.8\pm\:0.2$	$\:1.3\pm\:0.2$	$\:4.4\pm\:0.3$
823	Comb 𝛿_ip_CCH + 𝛿_ip_CCC *	$\:2.1\pm\:0.5$	2.5 ± 0.6	$\:1.5\pm\:0.3$	$\:0.8\pm\:0.2$	$\:3.0\pm\:0.2$
764	Fund 𝛿_oop_CH *^/^**	$\:3.9\pm\:0.6$	1.4 ± 0.2	$\:0.8\pm\:0.1$	$\:1.4\pm\:0.2$	$\:2.3\pm\:0.1$
534	Comb 𝜏CC + 𝛿_oop_CH *^/^**	$\:2.2\pm\:0.6$	2.4 ± 0.6	$\:1.4\pm\:0.3$	$\:0.8\pm\:0.2$	$\:0.42\pm\:0.03$
Weighted average values		$\:2.3\pm\:0.1$	$\:1.8\pm\:0.1$	$\:1.0\pm\:0.1$	$\:0.88\pm\:0.04$	$\:0.83\pm\:0.02$

^* 85 ; ** DFT calculations (this work)^.

**Fig. 5:**
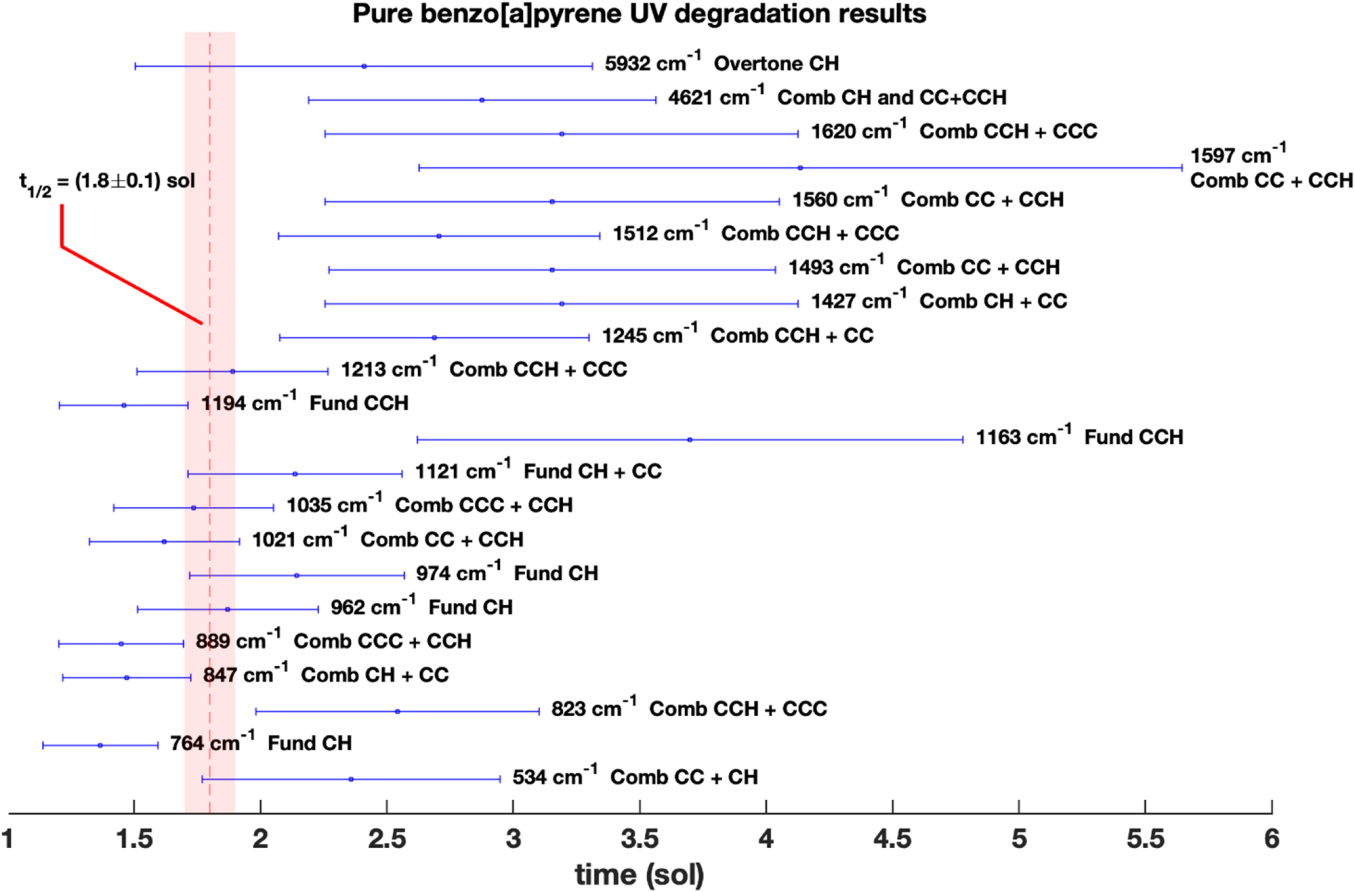
Pure benzo[a]pyrene half-lifetime degradation values$\:\:\left(sol\right)$ relative to the Jezero UV flux for the molecular bands assigned to the ring and CH groups. The vertical red dashed line is the weighted average half-lifetime. Legend: CC and CH represent carbon-carbon and carbon-hydrogen vibrations, respectively. CCC represents a vibration involving three carbon atoms. Meanwhile, CCH represents a vibration involving two carbon atoms and one hydrogen atom.

As observed in Table 2 and Fig. 5, twenty-three molecular bands assigned to the ring and CH vibrations show a degradation in the pure molecule as a consequence of UV irradiation (See Supplementary Figure S5). At the bottom of Table 2 weighted average values are reported.

In particular, bands with wavenumbers less than $\:\sim\:1200\:{cm}^{-1}$ show lower half-lifetimes, thus a more vulnerable vibrational modes under UV stress. The vibrational modes in this part of the spectrum correspond to specific vibrations like torsion or bending of CH and CC, as well as intra-molecular motions. Moreover, pure benzo[a]pyrene bands in the mid-spectrum region, between $\:800\:{cm}^{-1}$ and $\:1300\:{cm}^{-1}$, show higher DP, reaching more than $\:10\%$ of area degradation, and also present a faster degradation. All the benzo[a]pyrene fundamental bands that show a degradation behavior fall in this spectrum region, i.e. $\:1245\:{cm}^{-1}$ (Fundamental 𝛿_ip_CCH), $\:1194$ $\:{cm}^{-1}$ (Fundamental 𝛿_ip_CCH), $\:1163\:{cm}^{-1}$ (Fundamental 𝛿_ip_CCH), $\:1121$ $\:{cm}^{-1}$ (Fundamental 𝛿_ip_CCH), $\:1111$ $\:{cm}^{-1}$ (Fundamental 𝛿_ip_CCH ) and $\:974\:{cm}^{-1}$ (Fundamental 𝛿_oop_CH). Energetically stronger vibrational modes like CH and CC stretching, instead, fall at higher wavenumbers, and have greater degradation half-lifetimes, thus lower degradation rates, which means higher resistance to UV exposure.

Among the benzo[a]pyrene bands analyzed, the fastest degradation is observed for the $\:764$ $\:{\:cm}^{-1}$ band assigned to the fundamental 𝛿_oop_CH vibration, with an half-lifetime of $\:{t}_{1/2}^{764}=\left(1.4\pm\:0.2\right)\:sol$ and cross section $\:{{\upsigma\:}}_{764}=\left(1.4\pm\:0.2\right)\:{10}^{-20}{cm}^{2}$ relative to Jezero UV flux (Table 2; Fig. 5).

Overall, the weighted mean half-lifetime value for all the benzo[a]pyrene (bap) bands analyzed is $\:{t}_{1/2}^{\overline{bap}}=\left(1.8\pm\:0.1\right)\:sol$ with a cross section of $\:{{\upsigma\:}}^{\overline{bap}}=\left(0.88\pm\:0.04\right)\cdot\:{10}^{-20}\:{cm}^{2}$ relative to the Jezero UV flux (Table 2; Fig. 5). Noteworthy, the weighted average amount of molecule degraded at the end of the UV experiment after reaching the plateau is $\:{\text{D}\text{P}}^{\overline{bap}}=\left(0.83\pm\:0.02\right)\:\%$.

On the other hand, for the benzo[a]pyrene adsorbed on hydrated magnesium sulfate, no significant degradation is observed for the entire duration of our UV irradiation experiment, suggesting a photoprotective behavior also for the benzo[a]pyrene molecule. For other bands, however, signals of change in the adjacent continuum which are not associated with band degradation can be observed.

**Fig. 6:**
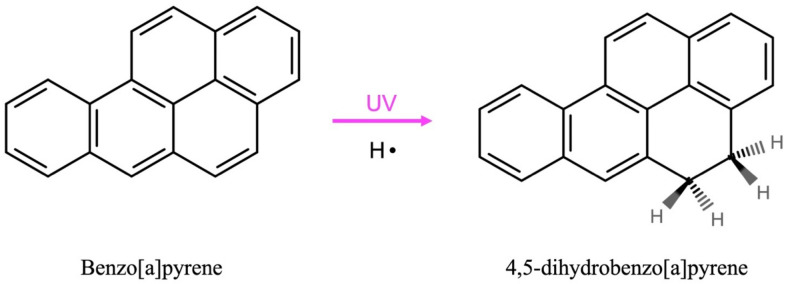
Suggested 4,5-dihydrobenzo[a]pyrene photoproduct formation from benzo[a]pyrene through radical processes due to the UV irradiation exposure.

Using LC-LEI-HRMS, the irradiated pure benzo[a]pyrene sample revealed the formation of dihydrobenzo[a]pyrene as a photoproduct. The latter presents three main isomers according to the position of the saturation in the structure: the 4,5 or 7,8 or 9,10-dihydrobenzo[a]pyrene. The NIST library reports the MS spectra of 4,5 and 7,8-dihydrobenzo[a]pyrene but lacks the one for the 9,10-isomer. A comparison of the experimental spectrum with the spectra reported in the NIST library shows a greater similarity with the 4,5-isomer formed due to hydrogenation of the CC double bond in the 4,5-position. Its tentative radical chemical formation pathway is showed in Fig. 6.

This compound was not detected in the pre-irradiated sample of benzo[a]pyrene, confirming that it is generated as a result of the UV exposure. In addition, the same photoproduct was found in the irradiated 1 wt% benzo[a]pyrene adsorbed on the hydrated magnesium sulfate. Instead, no photoproduct was detected in the non-irradiated 1 wt% benzo[a]pyrene adsorbed on hydrated magnesium sulfate, confirming again that it’s formed due to the UV radiation. The photoproduct, dihydrobenzo[a]pyrene, was present in higher quantity in the pure molecule irradiation experiment compared to the irradiated sample where benzo[a]pyrene was adsorbed on the hydrated magnesium sulfate. A semi-quantitative result of the amount of photoproduct formed give us 0.048 wt% of dihydrobenzo[a]pyrene for the pure molecule experiment and 0.027 wt% of dihydrobenzo[a]pyrene from the total of benzo[a]pyrene in the sample. This observation together with the IR half-lifetimes results, suggests that the mineral photoprotects the benzo[a]pyrene from UV radiation.

### Implications for the NASA Mars 2020 mission at Jezero crater

Perseverance abrades the surface and analyzes subsurface material with its payload instruments, like SuperCam and SHERLOC, to determine the mineralogical and organic content of the rocks. For operational reasons, these abraded patches remain exposed to ambient UV for at least $\:1\:sol$ before proximity science measurements, which means that any organic material in the abrasion patches must be stable under UV radiation for at least one sol in order to be detected.

SHERLOC found interesting Raman features in spectral regions relevant to organics, co-located with sulfate minerals, in the Quartier abrasion of the Issole outcrop in the Jezero crater floor^22^. Interestingly, these features remained unaltered for 11 sols, from sol 293, when they were first detected, to sol 304, when their detection was reconfirmed^22^. Comparison with laboratory data indicate that these features could be due to PAHs^23^. This would hold true only if those organic compounds in sulfates can withstand UV exposure for at least 11 sols.

Our UV irradiation experiments indicate that PAHs like 2,6-dihydroxynaphthalene and benzo[a]pyrene can be detected even after 11 sols of exposure to Martian ambient UV if adsorbed on magnesium sulfate thanks to the photoprotective properties of magnesium sulfate, in contrast to the pure molecules which would degrade faster, with weighted average half-lifetimes of $\:\left(10\pm\:1\right)\:sol$ and $\:\left(1.8\pm\:0.1\right)\:sol$, respectively (Tables 1 and 2; Figs. 3 and 4).

These results suggest a photoprotective behavior of hydrated magnesium sulfate toward the two PAHs, and corroborates the hypothesis that the SHERLOC Raman signals observed spatially co-located with sulfates in Quartier might be due to PAHs.

Moreover, this work shows that the SuperCam IR in-situ analysis (spectral range $\:7700-3850$ $\:{cm}^{-1}$, $\:1.3-2.6$ $\:\mu\:m$) can contribute to organics detection. In fact, the $\:5932$ $\:{cm}^{-1}$ ($\:5939$ $\:{cm}^{-1}$ after the molecular adsorption), $\:4621$ $\:{cm}^{-1}$ and $\:4496$ $\:{cm}^{-1}$ benzo[a]pyrene bands and most of the 2,6-dihydroxynaphthalene bands are still present in the SuperCam IR range after their adsorption on hydrated magnesium sulfate, meaning that also SuperCam could potentially detect such organics in magnesium sulfate if present at $\:>1$ wt.$\:\%$ concentrations (See Supplementary Table S4 for the specific wavelengths of the organic bands in the SuperCam spectral range). Finally, following molecular adsorption, the water-related absorption bands of the mineral exhibit spectral changes that may be attributed to the presence of organic compounds. In particular, the magnesium sulfate bands at $\:5161$ $\:{cm}^{-1}$ ($\:1.9\:\mu\:m$), corresponding to the combination of water OH stretching and bending, and at $\:6812$ $\:{cm}^{-1}$ ($\:1.5\:\mu\:m$), assigned to the $\:1$^st^ overtone of the water OH stretching, fall within the SuperCam spectral range.

## Conclusions

This work reports the IR characterization and UV irradiation of Martian analog samples obtained by adsorbing two PAHs, i.e. 2,6-dihydroxynaphthalene (2,6-dhn) and benzo[a]pyrene, on hydrated magnesium sulfate in water followed by desiccation.

The vibrational spectroscopy characterization of these samples shows that most of the characteristic vibrational bands of the pure molecules are either not present, or strongly attenuated, or undergo shifts, when the molecules are adsorbed on the mineral. Especially for the benzo[a]pyrene experiments the dilution of the organic compound within the mineral matrix (1 wt%) has played a role. Moreover, molecular adsorption induces spectral variations in the water-associated absorption bands of the mineral, which may be indicative of the presence of organic compounds. This highlights the importance of acquiring databases of infrared spectroscopic features for organo-mineral complexes to support detection of organics on Mars.

IR characterization shows PAHs-mineral interaction as demonstrated by molecular band shifts in the post adsorption spectra. Specifically, the shifts suggest that 2,6-dhn interacts with hydrated magnesium sulfate through OH and CH functional groups, while benzo[a]pyrene mainly through CH functional groups. In particular, for 2,6-dhn, splitting of the fundamental 𝛿_ip_CH and 𝛿_ip_OH band at $\:1150$ $\:{cm}^{-1}$ and fundamental 𝛿_oop_CH band at $\:656$ $\:{cm}^{-1}$ are recorded when adsorbed on the mineral. UV irradiation experiments allowed us to assess the stability of these PAHs, both in the pure state and adsorbed on hydrated magnesium sulfate once exposed to the Martian UV radiation environment. In particular, the pure 2,6-dhn shows a photodegradation with a weighted average half-lifetime of $\:\left(10\pm\:1\right)\:sol$ relative to Jezero UV flux. On the other hand, the pure benzo[a]pyrene shows a photodegradation with a lower weighted average half-lifetime of $\:\left(1.8\pm\:0.1\right)\:sol$. Moreover, the amount of molecule degraded are $\:{\text{D}\text{P}}^{\overline{\text{2,6}-dhn}}=\left(0.55\pm\:0.02\right)\%$ for 2,6-dhn and $\:{\text{D}\text{P}}^{\overline{bap}}=\left(0.83\pm\:0.02\right)\%$ for benzo[a]pyrene.

In addition, greater photostability is observed for both PAHs when adsorbed on the hydrated magnesium sulfate. This photostability is recorded despite a Jezero UV flux exposure of $\:64$ sols for 2,6-dhn and $\:25$ sols for benzo[a]pyrene ($\:48\:sols$ and $\:19\:sols$ considering the predicted Patel’s UV flux).

Using LC-LEI-HRMS, it was possible to identify the presence of photoproducts resulting from the exposure to UV radiation. During the irradiation of 2,6-dhn, 6-methoxy-2-naphthol was formed, whereas no photoproducts were detected when 2,6-dhn was adsorbed on magnesium sulfate. In contrast, the photoproduct dihydrobenzo[a]pyrene was detected both after irradiation of pure benzo[a]pyrene and when adsorbed on magnesium sulfate, in the latter case in approximately half the amount. These photoproduct analyses also suggest a photoprotective effect of this mineral.

In both the 2,6-dhn and benzo[a]pyrene case studies, spectral changes were observed in the water-associated IR bands of hydrated magnesium sulfate after organic adsorption for the $\:6812$ $\:{cm}^{-1}$ ($\:1.5\:\mu\:m$), $\:5161$ $\:{cm}^{-1}$ ($\:1.9\:\mu\:m$) and $\:3583$ $\:{cm}^{-1}$ ($\:2.8\:\mu\:m)$. These changes are inconsistent with kieserite, as confirmed by comparison with reference spectra of kieserite and hexahydrite (Supplementary Figures S1 and S2). Therefore, dehydration to kieserite can be excluded. The observed features are more plausibly explained by the coexistence of multiple hydration states of magnesium sulfate, with hydration levels higher than kieserite. All these results point to the photoprotective behavior of magnesium sulfate hydrate towards these two PAHs, corroborating the key-role of sulfates in the preservation of possible Martian organic molecules. In addition, the SHERLOC Raman signals in association with sulfate detected in Quartier could potentially arise from PAHs. Moreover, the photoprotective properties of magnesium sulfate may explain why the strongest SHERLOC Raman signals have been observed co-located with sulfates rather than other minerals that may photocatalyzed organic degradation prior to the SHERLOC analysis. This raises concerns about potential biases in detecting organics in other minerals and the consequent potential underestimation of the astrobiological significance of collected samples based on delayed SHERLOC analysis of abraded patches. These limitations of in-situ investigations underscore the necessity of Mars Sample Return (MSR) to accurately assess the organic content of Martian samples gathered by Perseverance. Therefore, analyses in terrestrial laboratories will be crucial as we will be able to use more highly-targeted (higher magnification and long-working microscope objectives), high-resolution spectroscopy (with beams that can reach $\:\sim\:1-2$ $\:\mu\:m$ in diameter) and other techniques to detect organics.

Beyond the Mars 2020 mission, these studies are relevant also to other Martian rover exploration missions such as Curiosity, the future ESA ExoMars/Rosalind Franklin rover mission and orbital remote sensing observations. In particular, Curiosity rover is about to begin exploring the strata in Mount Sharp with orbital spectral absorptions interpreted to indicate an enrichment of sulfate minerals, like a magnesium one, at different hydration states.

In addition, since sulfates are widespread also on other rocky bodies in the Solar System such as the icy moons of gas giants (e.g. Europa or Io moons), this laboratory analog work can support the future Europa Clipper mission as well as the interpretation of the data acquired by MIRI instrument of the James Webb Telescope (JWST).

## Methods

### Mars analog sample Preparation

The Mars analog samples were prepared using epsomite (MgSO_4_$\:\cdot\:$7H_2_O) as the mineral phase (purity > 99.5%, Sigma Aldrich), that was actually characterized as a mixture of epsomite and hexahydrite thanks to an infrared spectroscopic assessment, and two polycyclic aromatic hydrocarbons (PAHs): 2,6-dihydroxynaphthalene (98% purity, Sigma Aldrich) and benzo[a]pyrene ($\:\ge\:$96% purity, Sigma Aldrich). To replicate the physico-chemical interactions that might have occurred between organic molecules and minerals in early Martian aqueous environments, the samples were prepared by mixing the mineral powder in an aqueous suspension containing the PAH molecules. The mixture was continuously agitated on a tube plate for 24 h, following the procedure outlined by Fornaro et al.^2^. Specifically, a mineral concentration of 140 g/L, with 10 wt% of 2,6-dihydroxynaphthalene and 1 wt% of benzo[a]pyrene, was employed. A lower concentration is used for benzo[a]pyrene because at 1 wt%, molecular bands intense enough to be analyzed in this experiment are already observed in the IR spectrum. This organic concentration is significantly higher than what detected on Mars by the Curiosity rover, but was selected to ensure the detection of strong molecular IR bands and facilitate the monitoring of degradation kinetics during UV exposure experiments. The suspensions were then dried in an oven at mild conditions (40 °C) to mimic a desiccation event that could have occurred on Mars in the past. A control sample (blank) of epsomite was also prepared using the same method, but without the addition of PAHs.

### Infrared (IR) characterization

The characterization of the samples was performed by DRIFTS - Diffuse Reflectance Infrared Fourier Transform Spectroscopy. DRIFTS measurements were carried out at INAF - Astrophysical Observatory of Arcetri using a Bruker VERTEX 70v FTIR instrument equipped with a Praying Mantis^™^ Diffuse Reflection Accessory (Harrick DRIFT), using a Globar source, DigiTech DLaTGS detector, KBr beamsplitter. Spectra were acquired using $\:100$ scans of the interferometer with a resolution of $\:4$ $\:{cm}^{-1}$ in the wavenumber range $\:8000-400$ $\:{cm}^{-1}$ $\:(1.25-25\:\mu\:m)$. The Praying Mantis^™^ in the interferometer sample compartment was saturated with nitrogen to reduce atmospheric contamination during the IR measurements, and to prevent oxidation during irradiation experiments.

### Ultraviolet (UV) irradiation experiments

The experimental setup consists of a Newport Oriel 300 W Xenon discharge lamp that produces an emission spectrum corresponding to blackbody emission at 5800 K with superimposed Xe emission lines interfaced with the Bruker VERTEX 70v interferometer. An $\:800\:\mu\:m$ fiber optic was placed directly inside the interferometer sample compartment to irradiate the sample in situ during the experiment. The optical fiber transmits approximately $\:100$ $\:mW\:$power to the sample in the 200–2500 nm spectral range. See Supplementary Note “UV lamp details” with Supplementary Figure S6, Supplementary Figure S7 and Supplementary Figure S8 for more details. With this configuration, the irradiated spot of the sample has an area of $\:7.07\:{mm}^{2}$ and the UV lamp flux focused on the sample is $\:{{\Phi\:}}_{Lamp}=2.75\cdot\:{10}^{17}\:photons\cdot\:{s}^{-1}\cdot\:{cm}^{-2}$ in the spectral range $\:200-400\:nm$, measured through a Spectro 320 monochromator scanning spectrometer (Instrument System). Infrared spectra were recorded at regular intervals during UV irradiation to monitor the photodegradation: a total time of $\:10800\:seconds$ ($\:3$ hours) for 2,6-dihydroxynaphthalene and a total time of $\:4200\:seconds$ ($\:1.16$ hours) for benzo[a]pyrene. A longer irradiation time for 2,6-dihydroxynaphthalene was chosen because of the higher stability of the molecule. Specifically, infrared spectra were recorded initially every five seconds to monitor the quickest changes usually happening at the beginning of the UV irradiation. Then, the time intervals between infrared measurements increased up to tens of minutes. The total irradiation time of the pure PAH experiment is the same as that of PAH adsorbed on magnesium sulfate. This procedure allowed us to follow the degradation process in real time and the possible formation of new species by observing changes in the infrared spectroscopic characteristics^69^. The degradations of the same molecular bands in the case of the pure molecule and when the molecules are adsorbed on the mineral were compared to investigate the photoprotective/photocatalytic properties of the mineral.

The relative areas $\:A$ of the same molecular bands for pure molecule and molecule adsorbed on epsomite were calculated for each spectrum using MATLAB R2022a software (MATLAB Version: 9.12.0.1975300 Update 3). The ratio $\:A\left(t\right)/A\left(0\right)$ was plotted versus the irradiation time $\:t$, where $\:A\left(t\right)$ is the area of the band at a given irradiation time $\:t$, proportional to the number of molecules at that time, and $\:A\left(0\right)$ is the area of the band before the irradiation process, proportional to the initial number of molecules at time $\:t=0$. A first-order kinetics function was used to fit the experimental data:

$$\:\frac{A\left(t\right)}{A\left(0\right)}=B{e}^{-\beta\:t}+C$$

where $\:B$ is the fraction of molecules that interact with UV radiation, $\:\beta\:$ is the degradation rate and $\:C$ is the fraction of molecules that do not interact with UV radiation because of their position deep in the solid sample. UV radiation, in fact, can only penetrate to a depth of a few micrometers, as opposed to IR radiation. From the degradation rate $\:\beta\:$, obtained from the fit, it is possible to calculate the half-life $\:{t}_{1/2}$, or the time required to destroy $\:50\:\%$ of the initial number of molecules, using the following formula:

$$\:{t}_{\frac{1}{2}}=\frac{\text{ln}2}{\beta\:}$$

From $\:\beta\:$ it is also possible to calculate the UV destruction cross section $\:\sigma\:$ that represents the probability of interaction between UV radiation and molecule:

$$\:\sigma\:=\frac{\beta\:}{{{\Phi\:}}_{Lamp}}$$

where, $\:{{\Phi\:}}_{Lamp}$ is the total incident UV lamp flux mentioned above. The degradation percentage (DP%) value is the $\:B$ parameter in the first-order kinetics function used in the fit calculation and represents how much band area has actually degraded in percentage terms. Finally, to estimate the survivability of this molecule on Mars, half-lives were scaled on two different Martian UV flux values: the first one is the Patel et al.^12^ Martian UV flux in the $\:190-325\:nm$ spectral region, assuming dust free atmosphere at the noontime equator, that is $\:{\varPhi\:}_{Mars}^{Patel}=1.4\cdot\:{10}^{15}\:photons\cdot\:{s}^{-1}\cdot\:{cm}^{-2}$, good for a general purpose evaluation; the second one is the Jezero Crater UV flux $\:{\varPhi\:}_{Mars}^{Jezero}=8.1\cdot\:{10}^{14}\:photons\cdot\:{s}^{-1}\cdot\:{cm}^{-2}$, more accurate for the Mars 2020 Perseverance purpose. The latter one is calculated for the same spectral region using the radiative transfer model COMIMART^80^, which includes state-of-the-art dust radiative properties^81,82^, and is fed with Mastcam-Z opacities^83,85^. Specifically, the annual total number of photons per unit surface area was obtained using the simulations for one full Mars Year (from sols 16 to 684) and this value was divided by the effective time of UV irradiation (Sun-time).

### Computational assignments

Quantum chemical calculations were performed for the target molecules to simulate their IR spectra and support the analysis of the experimentally acquired signals in terms of normal modes of vibration. For 2,6-dihydroxynaphthalene the double-hybrid functional DSDPBEP86^86^ in conjunction with the jun-cc-pVTZ basis set^87^, and the PW6B95^88^ meta-hybrid functional coupled with the jul-cc-pVDZ basis set were employed because of their accuracy in the prediction of IR transition frequencies and intensities^89–91^. In the case of benzo[a]pyrene, the B3LYP density functional^92^ in conjunction with the 6-31G(*d*)^93^ basis set was used to both limit the computational cost due to the larger molecular size, and to the fact that, for this molecule, the jul-cc-pVDZ basis set led to unreliable anharmonic corrections. All density functionals were augmented for dispersion interactions through the DFT-D3 scheme^94^ with Becke-Johnson damping^95^. Geometry optimizations were carried out at first using very tight convergence criteria and then analytical hessians and dipole moment first derivatives were evaluated at the equilibrium molecular structures and, from these, IR harmonic frequencies and intensities were obtained. For the calculations carried out using the PW6B95 and B3LYP density functionals, cubic and semi-diagonal quartic force constants as well as second- and third-derivatives of the dipole moment surface were computed by numerical differentiation of analytic quadratic force constants and dipole moment first derivatives, respectively. These take into account the effects of mechanical- and electrical-anharmonicity and hence are required to derive transition frequencies, and intensities beyond the double-harmonic approximation within the framework of second-order vibrational perturbation theory (VPT2)^96^. In order to obtained more accurate spectral simulations, and take advantage of the harmonic force field computed at DSDPBEP86/jun-cc-pVTZ level, a hybrid force field^97^ was worked out for 2,6-dihydroxynaphthalene. Specifically, DSDPBEP86-D3/jun-cc-pVTZ harmonic frequencies and intensities were supplemented by anharmonic contributions calculated at the PW6B95-D3/jul-cc-pVDZ level. Generalized VPT2^98,99^ was then adopted to overcome the problem of anharmonic cubic and quartic interactions. More in detail, resonant terms were first identified and removed from the perturbative equations and then, the interaction was treated in a second step of lower dimensionality by setting up and diagonalizing the proper resonance matrix^97^.

### LC-LEI-HRMS investigation

LC-MS analyses were performed using an Agilent 1260 Infinity II HPLC coupled to an Agilent 7250 GC/QTOF through a Liquid Electron Ionization (LEI) interface^100,101^. LC separation was achieved using an Acquity UPLC BEH C18 (1.7 μm, 1 × 100 mm) column at a flow rate of 0.1 mL/min, with a gradient from 5% Solvent B at 0 min to 100% Solvent B at 19 min, then held constant until 26 min (Solvent A: H₂O with 0.1% formic acid; Solvent B: ACN with 0.1% formic acid). A 2 µL sample was injected into the chromatographic system, and 500 nL/min was directed into the EI ion source via the LEI interface. The vaporization microchannel temperature was set to 400 °C to optimize the vaporization of target analytes.

Pure and photo-oxidized standards of 2,6-dhn and benzo[a]pyrene were solubilized in acetonitrile and tetrahydrofuran, respectively. Organo-mineral samples were extracted weighing an aliquot of 5 mg in a glass vial and adding 400 µL of solvent (acetonitrile and tetrahydrofuran for 2,6-DHN and benzo[a]pyrene, respectively). Successively, the mixture was agitated with a vortex for 5 min, sonicated for 2 min and then agitated with a vortex again for 5 min. Finally, the mixture was filtrated with a 0.2 μm PTFE syringe filter to remove the mineral and was used for LC-MS analyses. This extraction protocol allows the recovery of 100% of analyte from the mineral.

MS acquisitions were performed at 70 eV ionization energy in full-scan mode within a mass range of 83–600 m/z. Additionally, further analyses were conducted at a lower ionization energy of 15 eV to provide better qualitative information on high-mass fragments of unknown substances in the samples. Unknown compounds were identified through NIST library database searches and comparison with the experimental EI mass spectra obtained.

Finally, the semi-quantitative analyses of the photoproducts were performed calibrating the instrumental response of the samples studied with analyses of standard solutions at the desired concentrations (See Supplementary Figure S9 and Supplementary Figure S10). Validation of the method demonstrated a good intraday reproducibility with a relative standard deviation < 8% and limits of detection from the minerals of 80 ng/mg and 10 ng/mg for 2,6-dhn and benzo[a]pyrene, respectively.

## Supplementary Information

Below is the link to the electronic supplementary material.

Supplementary Material 1

## Data Availability

Data is provided within the manuscript or supplementary information files.
