# Supplementary material for: Photostability of polycyclic aromatic hydrocarbons in hydrated magnesium sulfate under Martian ultraviolet irradiation to assist organics detection on Mars
Source: Sci Rep. 2025 Nov 18;15:40484. doi: 10.1038/s41598-025-24253-8 (PMC12627640; doi:10.1038/s41598-025-24253-8)
Supplement: Supplementary file 1 — Supplementary Material 1 [file 41598_2025_24253_MOESM1_ESM.docx]

**Supplementary materials**

**-**

**Photostability of polycyclic aromatic hydrocarbons in hydrated magnesium sulfate under Martian ultraviolet irradiation to assist organics detection on Mars**

**Table S1 – IR bands detectable both in pure pure 2,6-dihydroxynaphthalene spectrum and pure 2,6-dihydroxynaphthalene adsorbed on hydrated magnesium sulfate, with the vibrational mode assignment and intensity (w = weak; m = medium; s = strong), along with the wavenumber shifts with respect to the pure molecule.**

| 2,6-dihydroxynaphthalene vibrational mode | Pure 2,6-dihydroxynaphthalene | | $\boldsymbol{10}$wt.%  2,6-dihydroxynaphthalene on hydrated magnesium sulfate | | |
| --- | --- | --- | --- | --- | --- |
|  | Wavenumber [cm^-1^] | Intensity | Wavenumber [cm^-1^] | Intensity | Shift [cm^-1^] |
| 𝜈CH + 𝜈CH** | 6110 | w |  |  |  |
| 𝜈CH + 𝜈CH** | 5960 | s | 5960 | m |  |
| 𝛿_ip_CH and 𝛿_ip_OH + 𝜈OH** | 4812 | w | 4812 | w |  |
| 𝛿_ip_CH + 𝜈OH** | 4765 | w | 4765 | w |  |
| 𝜈CH and 𝛿_ip_OH and 𝛿_ip_CH + 𝜈CH** | 4682 | w | 4682 | w |  |
| 𝜈CH and 𝛿_ip_OH and 𝛿_ip_CH + 𝜈CH** | 4657 | s | 4657 | m |  |
| 𝜈CH and 𝛿_ip_OH + 𝜈CH** | 4636 | w | 4636 | w |  |
| 𝛿_ip_CH and 𝜈CH and 𝛿_ip_OH + 𝜈CH** | 4583 | w | 4581 | w |  |
| 𝛿_ip_CH and 𝜈CH and 𝜈OH + 𝜈CH** | 4560 | w | 4560 | w |  |
| 𝛿_oop_CH + 𝜈OH** | 4536 | w | 4536 | w |  |
| 𝛿_oop_CH + 𝜈OH** | 4515 | w | 4513 | w |  |
| 𝛿_ip_CH and 𝛿_ip_OH + 𝜈CH** | 4434 | m | 4428 | m | -5 |
| 𝛿_ip_CH and 𝛿_ip_OH + 𝜈CH * | 4326 | m | 4328 | w |  |
| 𝜈C-OH and ring breathing + 𝜈CH * | 4309 | s | 4309 | w |  |
| 𝛿_ip_CH + 𝜈CH** | 4282 | w |  |  |  |
| 𝛿_ip_OH + 𝜈CH** | 4263 | w |  |  |  |
| 𝛿_ip_CH and 𝛿_ip_OH + 𝜈CH** | 4216 | m | 4216 | w |  |
| 𝛿_ip_CH + 𝜈CH * | 4189 | m | 4189 | w |  |
| 𝛿_ip_CH + 𝜈CH** | 4166 | w |  |  |  |
| 𝛿_ip_CH + 𝜈CH** | 4156 | w |  |  |  |
| 𝛿_ip_CH + 𝜈CH** | 4143 | w |  |  |  |
| 𝛿_ip_C-OH + 𝜈OH** | 4064 | w |  |  |  |
| 𝛿_ip_CH and 𝜈CH + 𝜈CH** | 4031 | w | 4031 | w |  |
| 𝛿_ip_C-OH + 𝜈OH** | 4014 | m | 4010 | w |  |
| 𝛿_ip_CH and 𝜈CH + 𝜈CH** | 3989 | w | 3987 | w |  |
| 𝛿_oop_OH + 𝜈OH** | 3977 | w | 3977 | w |  |
| 𝜈OH* | 3244 | s |  |  |  |
| 𝛿_ip_CH and 𝜈CH and 𝛿OH + 𝜈CH and 𝛿_ip_OH and 𝛿_ip_CH** | 3130 | w |  |  |  |
| 𝜈CH** | 3064 | w | 3064 | w |  |
| 𝜈C* | 3047 | w | 3049 | w |  |
| 𝜈CH** | 3001 | w | 3001 | w |  |
| 𝜈C-OH, ring breathing and 𝛿CH + 𝜈CH and 𝛿_ip_OH and 𝛿_ip_CH** | 2954 | w |  |  |  |
| 𝛿_ip_CH and 𝛿_ip_OH + 𝜈CH, 𝛿_ip_OH and 𝛿_ip_CH** | 2936 | w | 2936 | w |  |
| 𝛿_ip_CH, 𝛿_ip_OH and 𝜈CC + 𝛿_ip_CH, 𝜈CC and 𝛿OH** | 2899 | w | 2901 | w |  |
| 𝜈CH* | 2874 | m | 2878 | w |  |
| 𝛿_ip_CH, 𝛿_ip_OH and 𝜈CH + 𝜈CC, 𝛿_ip_CH and 𝛿OH** | 2802 | w | 2804 | w |  |
| 𝛿_ip_CH and 𝛿_ip_OH + 𝜈CC (adjacent to OH), 𝛿_ip_CH and 𝛿OH** | 2777 | w |  |  |  |
| 𝛿ipCH and 𝛿ipOH** | 2764 | w | 2764 | w |  |
| 𝛿ipCH + 𝜈CH and 𝛿ipOH** | 2731 | m | 2733 | w |  |
| 𝛿_ip_CH and 𝛿_ip_OH + 𝛿_ip_CH, 𝜈CC (adjacent to OH) and 𝛿_ip_OH** | 2706 | m | 2706 | w |  |
| 𝜈C-OH, ring breathing and 𝛿CH + 𝛿_ip_CH, 𝛿_ip_ OH and 𝜈CC** | 2687 | w | 2685 | w |  |
| 𝛿_ip_OH and 𝛿_ip_CH + 𝛿_ip_CH, 𝛿OH and 𝜈CH** | 2655 | w | 2654 | w |  |
| 𝛿_ip_CH + 𝛿_ip_CH, 𝜈CH and 𝛿OH** | 2636 | w | 2635 | w |  |
| 𝛿_ip_OH + 𝛿_ip_CH, 𝛿_ip_OH and 𝜈CH** | 2606 | w | 2606 | w |  |
| 𝜈C-OH, ring breathing and 𝛿CH** | 2565 | w | 2567 | w |  |
| 𝛿_ip_CH and 𝛿_ip_OH + 𝜈C-OH, ring breathing and 𝛿CH** | 2548 | s | 2542 | m | -6 |
| 𝛿_ip_CH and 𝛿_ip_OH + 𝛿_ip_CH and 𝛿_ip_OH** | 2448 | w | 2444 | w |  |
| 𝛿_ip_CH and 𝛿_ip_OH + 𝛿_ip_CH** | 2430 | w | 2428 | w |  |
| 𝛿_ip_CH** | 2268 | w | 2268 | w |  |
| 𝛿_ip_ ring deformation + 𝜈CH, 𝛿_ip_OH and 𝛿_ip_CH** | 2253 | w | 2255 | w |  |
| 𝛿_oop_CH + 𝜈C-OH, ring breathing and 𝛿CH** | 2235 | w | 2237 | w |  |
| 𝛿_ip_ ring deformation + 𝛿_ip_CH, 𝜈CH and 𝛿OH** | 2199 | m | 2203 | w |  |
| 𝛿_oop_CH + 𝛿_ip_CH, 𝛿_ip_OH and 𝜈CC** | 2185 | m | 2181 | w |  |
| 𝛿_ip_CH and 𝜈CH + 𝛿_ip_OH and 𝛿_ip_CH** | 2172 | w |  |  |  |
| 𝛿_oop_CH + 𝛿_ip_CH and 𝛿_ip_OH** | 2133 | w | 2135 | w |  |
| 𝛿_ip_CH and 𝛿_ip_C-OH + 𝜈CC, 𝛿_ip_OH and 𝛿_ip_CH** | 2122 | m | 2124 | w |  |
| 𝛿_ip_CH and 𝜈CC and 𝛿OH + ring deformation** | 2089 | m | 2091 | w |  |
| 𝛿_oop_CH + 𝛿_ip_CH and 𝛿_ip_OH** | 2083 | m | 2083 | w |  |
| 𝛿_ip_CH, 𝜈CC and 𝛿_ip_OH + ring deformation** | 2071 | w |  |  |  |
| 𝛿_ip_ ring deformation + 𝛿_ip_CH and 𝛿_ip_OH** | 2029 | w | 2029 | w |  |
| 𝛿_oop_CH + 𝛿_ip_CH** | 2006 | w | 2008 | w |  |
| 𝛿_ip_ C-OH + 𝜈CH, 𝛿_ip_OH and 𝛿_ip_CH** | 1994 | w | 1994 | w |  |
| 𝛿_oop_CH + 𝛿_ip_OH** | 1977 | m | 1977 | w |  |
| 𝛿_ip_CH + 𝛿_ip_CH** | 1965 | w | 1965 | w |  |
| 𝛿_ip_ ring deformation + 𝛿_ip_CH, 𝛿_ip_OH and 𝜈CH** | 1942 | w | 1944 | w |  |
| 𝛿_ip_CH and 𝜈CH** | 1933 | w | 1933 | w |  |
| 𝛿_oop_CH** | 1909 | s | 1909 | m |  |
| 𝛿_ip_ ring deformation + 𝜈C-OH, ring breathing and 𝛿CH** | 1873 | m | 1870 | w |  |
| 𝛿_ip_ ring deformation + 𝛿_ip_CH and 𝛿_ip_OH** | 1861 | m | 1861 | w |  |
| 𝛿_oop_OH + 𝛿_ip_CH, 𝜈CH and 𝛿_ip_OH** | 1842 | w | 1846 | w |  |
| 𝛿_ip_C-OH + 𝛿_ip_CH, 𝛿_ip_OH and 𝜈CH** | 1827 | w | 1827 | w |  |
| 𝛿_ip_CH and 𝛿_ip_ ring deformation** | 1815 | w | 1813 | w |  |
| 𝛿_ip_C-OH + 𝛿_ip_CH and 𝛿_ip_OH** | 1800 | w |  |  |  |
| 𝛿_oop_CH** | 1769 | w | 1769 | w |  |
| 𝛿_ip_ ring deformation + 𝛿_ip_CH and 𝛿_ip_OH** | 1749 | w | 1751 | w |  |
| 𝛿_ip_C-OH + 𝛿_ip_CH and 𝛿_ip_OH** | 1738 | w | 1737 | w |  |
| 𝛿_oop_CH** | 1724 | w | 1724 | w |  |
| 𝛿_ip_ ring deformation + 𝛿_oop_CH** | 1717 | s | 1715 | w |  |
| 𝛿_oop_OH + CH and 𝛿_ip_OH** | 1699 | w |  |  |  |
| 𝜈CH, 𝛿_ip_OH and 𝛿_ip_CH** | 1684 | w |  |  |  |
| 𝛿_ip_CH and 𝛿_ip_C-OH + 𝛿_ip_OH** | 1638 | s | 1638 | m |  |
| 𝛿_oop_CH** | 1620 | m | 1622 | m |  |
| 𝜈CC* | 1609 | s |  |  |  |
| 𝛿_oop_CH + 𝛿_ip_CH and 𝛿_ip_ ring deformation** | 1603 | m | 1603 | m |  |
| 𝜈CH and 𝛿_ip_OH** | 1585 | w | 1580 | w | -5 |
| 𝛿_ip_ ring deformation + 𝛿_ip_CH and 𝜈CH** | 1557 | m | 1560 | w |  |
| 𝛿_oop_CH + 𝛿_oop_CH** | 1541 | w | 1541 | w |  |
| 𝜈CC* | 1514 | s | 1514 | s |  |
| 𝛿_oop_CH + 𝛿_oop_CH** | 1474 | m | 1474 | w |  |
| 𝜈CO* | 1418 | s | 1418 | s |  |
| 𝜈CC* | 1377 | s | 1375 | w |  |
| 𝛿_ip_CH and 𝛿_ip_OH** | 1366 | m | 1364 | w |  |
| 𝛿_ip_ ring deformation** | 1350 | w | 1350 | w |  |
| 𝛿_ip_CO* | 1312 | s | 1312 | m |  |
| 𝛿_ip_CH* | 1281 | s | 1281 | w |  |
| 𝛿_ip_CH and 𝛿_ip_OH** | 1269 | s | 1269 | w |  |
| 𝛿_ip_CH* | 1236 | s | 1236 | m |  |
| 𝛿_ip_CH + 𝛿_ip_OH*/** | 1215 | m | 1215 | m |  |
| 𝛿_ip_OH and 𝛿_ip_CH** | 1150 | s | 1150 | m |  |
|  |  |  | 1138 | w | -12 |
| 𝛿_ip_CH** | 1121 | m | 1121 | m |  |
| 𝛿_ip_CH* | 1113 | s | 1113 | m |  |
| 𝛿_ip_C-OH + 𝛿_ip_CH** | 1074 | w |  |  |  |
| 𝛿_oop_CH + 𝛿_oop_CH** | 1065 | w |  |  |  |
| 𝛿_oop_OH + 𝛿_oop_CH** | 1040 | w |  |  |  |
| 𝛿_ip_C-OH + 𝛿_ip_ ring deformation** | 1013 | m | 999 | m | -14 |
| 𝛿_ip_CH and 𝜈CH** | 980 | w | 980 | w |  |
| 𝛿_oop_CH* | 957 | s | 957 | m |  |
| 𝛿_oop_CH** | 943 | s | 943 | m |  |
| 𝛿_oop_CH** | 905 | w | 905 | w |  |
| 𝛿_ip_C-OH + 𝛿_oop_CH** | 891 | w |  |  |  |
| 𝛿_ip_CO* | 870 | s | 872 | m |  |
| 𝛿_oop_CH** | 862 | s | 862 | m |  |
| 𝛿_ip_CH** | 854 | w | 853 | w |  |
| 𝛿_oop_CH** | 810 | s | 810 | m |  |
| 𝛿_oop_CH* | 733 | m | 743 | w | +10 |
| 𝛿_ip_ ring deformation** | 696 | w | 696 | m |  |
| 𝛿_oop_CH* | 689 | m | 689 | w |  |
| 𝛿_oop_CH** | 656 | m | 673 | w | +17 |
|  |  |  | 663 | w | +7 |
| Ring deformation* + 𝛿_oop_OH** | 611 | m | 606 | w | -5 |
| 𝛿_oop_CO* | 588 | m | 590 | w |  |
| 𝛿_ip_ ring deformation** | 575 | m | 573 | w |  |
| 𝛿_oop_OH + 𝛿_ip_OH** | 502 | w |  |  |  |
| 𝛿_oop_ ring deformation + 𝛿_oop_OH** | 488 | m | 490 | w |  |
| 𝛿_oop_OH + 𝛿_oop_ ring deformation** | 467 | m | 469 | w |  |

* ^1^

** DFT calculations, this work


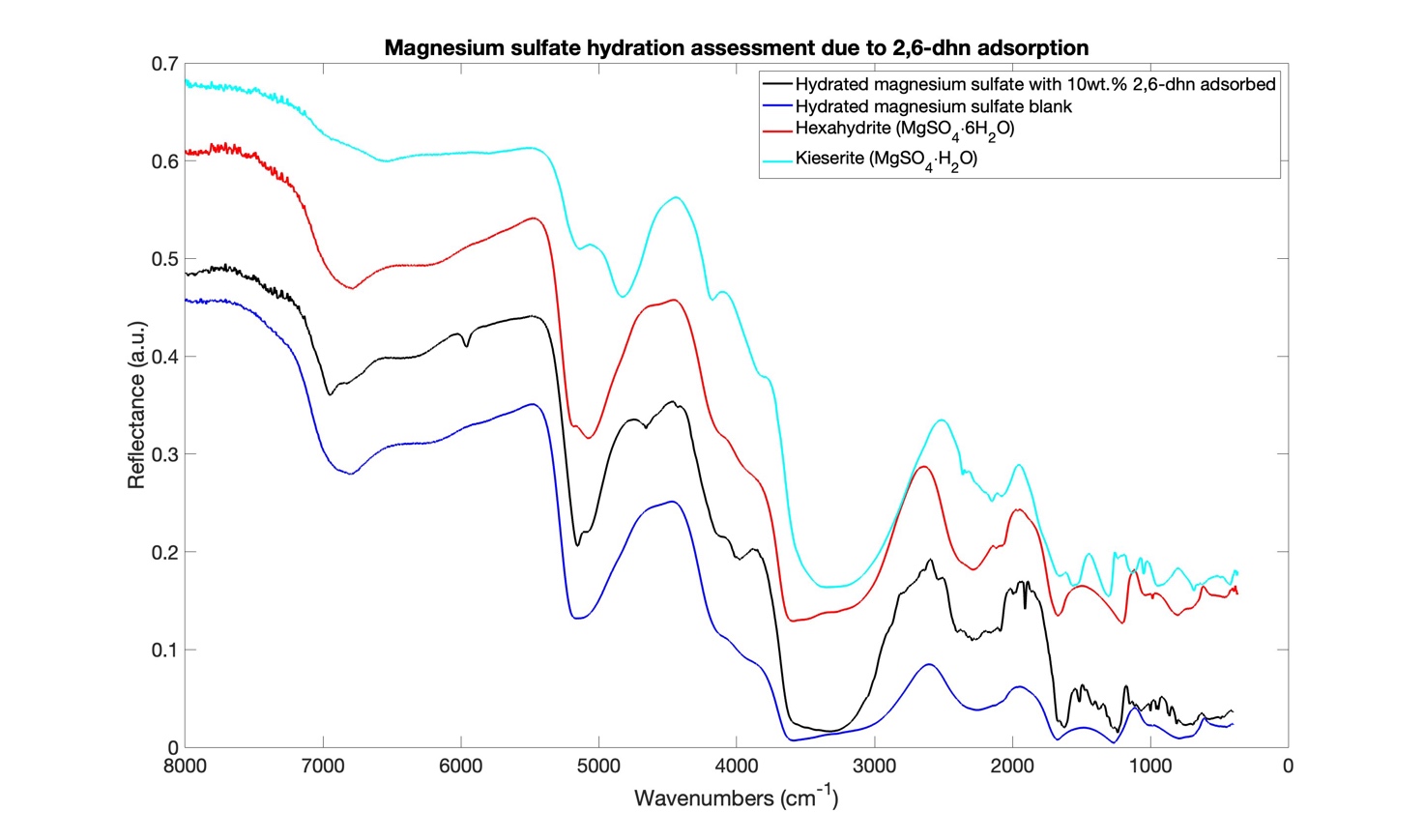


OH stretching band

Combination of water

OH stretching and OH bending

1st overtone of the

water OH stretching

**Figure S1**: IR spectra of hydrated magnesium sulfate with 10wt.% 2,6-dhn, hydrated magnesium sulfate blank, hexahydrite (MgSO4∙6H2O) and kieserite (MgSO4∙H2O) in order to assess the hydration state of the first two samples and investigate the organic role in the spectral changes.

**Table S2 – IR bands detectable both in pure pure benzo[a]pyrene spectrum and pure benzo[a]pyrene adsorbed on hydrated magnesium sulfate, with the vibrational mode assignment and intensity (w = weak; m = medium; s = strong), along with the wavenumber shifts with respect to the pure molecule.**

| benzo[a]pyrene vibrational mode | Pure benzo[a]pyrene | | $\boldsymbol{10}$wt.% benzo[a]pyrene on hydrated magnesium sulfate | | |
| --- | --- | --- | --- | --- | --- |
|  | Wavenumber [cm^-1^] | Intensity | Wavenumber [cm^-1^] | Intensity | Shift [cm^-1^] |
| 𝜈CH + 𝜈CH ^**^ | 6089 | m |  |  |  |
| Overtone 𝜈CH ^**^ | 5932 | s | 5939 | w | -7 |
| 𝜈CH + 𝜈CC and 𝛿_ip_CCH ^**^ | 4621 | s | 4621 | w |  |
| N.A. | 4565 | w |  |  |  |
| 𝜈CH + 𝜈CC and 𝛿_ip_CCH ^**^ | 4530 | w |  |  |  |
| 𝜈CH + 𝜈CC and 𝛿_ip_CCH ^*/**^ | 4496 | w | 4492 | w |  |
| 𝜈CH + 𝜈CC and 𝛿_ip_CCH ^**^ | 4477 | w |  |  |  |
| N.A. | 4467 | w |  |  |  |
| 𝜈CH + 𝜈CC and 𝛿_ip_CCH ^**^ | 4442 | w |  |  |  |
| N.A. | 4421 | w |  |  |  |
| 𝜈CH + 𝜈CC and 𝛿_ip_CCH ^**^ | 4359 | m |  |  |  |
| 𝜈CH + 𝜈CC and 𝛿_ip_CCH ^**^ | 4293 | w |  |  |  |
| N.A. | 4272 | w |  |  |  |
| N.A. | 4247 | w |  |  |  |
| N.A. | 4228 | w |  |  |  |
| N.A. | 4176 | m |  |  |  |
| 𝜈CH + 𝜈CC and 𝛿_ip_CCH ^**^ | 4155 | w |  |  |  |
| N.A. | 4135 | w |  |  |  |
| N.A. | 4112 | w |  |  |  |
| 𝜈CH + 𝜈CC and 𝛿_ip_CCH ^**^ | 4089 | w |  |  |  |
| 𝜈CH + 𝜈CC and 𝛿_ip_CCH ^**^ | 4072 | w |  |  |  |
| N.A. | 4021 | w |  |  |  |
| 𝜈CH + 𝛿_oop_CCC ^**^ | 3996 | w |  |  |  |
| 𝜈CH + 𝛿_oop_CCC ^**^ | 3985 | w |  |  |  |
| 𝜈CH + 𝛿_oop_CCC ^**^ | 3940 | w |  |  |  |
| 𝜈CH + 𝛿_oop_CCC ^**^ | 3931 | m |  |  |  |
| N.A. | 3530 | w |  |  |  |
| N.A. | 3518 | w |  |  |  |
| N.A. | 3489 | vw |  |  |  |
| N.A. | 3458 | vvw |  |  |  |
| N.A. | 3431 | vw |  |  |  |
| N.A. | 3410 | w |  |  |  |
| N.A. | 3385 | w |  |  |  |
| N.A. | 3366 | vw |  |  |  |
| N.A. | 3339 | vw |  |  |  |
| N.A. | 3300 | w |  |  |  |
| N.A. | 3252 | w |  |  |  |
| 𝜈CC and 𝛿_ip_CCH + 𝜈CC and 𝛿_ip_CCH ^**^ | 3235 | w |  |  |  |
| N.A. | 3206 | m |  |  |  |
| N.A. | 3181 | w |  |  |  |
| 𝜈CC and 𝛿_ip_CCH + 𝜈CC and 𝛿_ip_CCH ^**^ | 3150 | m |  |  |  |
| N.A. | 3094 | w |  |  |  |
| 𝜈CH ^**^ | 3074 | m |  |  |  |
| 𝜈CH ^**^ | 3046 | w |  |  |  |
| 𝜈CH ^*/**^ | 3034 | s |  |  |  |
| 𝜈CH ^**^ | 3003 | w |  |  |  |
| 𝜈CH ^**^ | 2986 | w |  |  |  |
| N.A. | 2974 | w |  |  |  |
| 𝜈CH ^**^ | 2936 | m | 2926 | w | -10 |
| N.A. | 2924 | w |  |  |  |
| N.A. | 2901 | w |  |  |  |
| 𝜈CC and 𝛿_ip_CCH + 𝛿_ip_CCH ^**^ | 2866 | m | 2855 | w | -11 |
| 𝜈CC and 𝛿_ip_CCH + 𝜈CC and 𝛿_ip_CCH ^**^ | 2833 | w |  |  |  |
| 𝛿_ip_CCH + 𝜈CC and 𝛿_ip_CCH ^**^ | 2797 | w |  |  |  |
| N.A. | 2772 | vw |  |  |  |
| 𝜈CC and 𝛿_ip_CCH + 𝛿_ip_CCH ^**^ | 2754 | w |  |  |  |
| N.A. | 2721 | vw |  |  |  |
| N.A. | 2698 | w |  |  |  |
| Overtone (𝜈CC and 𝛿_ip_CCH) ^**^ | 2691 | w | 2688 | w |  |
| N.A. | 2671 | vw |  |  |  |
| 𝛿_ip_CCH + 𝜈CC and 𝛿_ip_CCH ^**^ | 2654 | w |  |  |  |
| 𝛿_ip_CCH + 𝛿_ip_CCH ^**^ | 2639 | w | 2629 | w | -10 |
| N.A. | 2623 | w |  |  |  |
| N.A. | 2596 | vw |  |  |  |
| 𝜈CC and 𝛿_ip_CCH + 𝛿_ip_CCH ^**^ | 2569 | w | 2569 | w |  |
| 𝜈CC and 𝛿_ip_CCH + 𝛿_ip_CCC ^**^ | 2557 | m |  |  |  |
| N.A. | 2519 | w |  |  |  |
| 𝜈CC and 𝛿_ip_CCH + 𝛿_ip_CCC and other weak transitions ^**^ | 2505 | m |  |  |  |
| N.A. | 2490 | vw |  |  |  |
| N.A. | 2471 | vw |  |  |  |
| N.A. | 2448 | vw |  |  |  |
| N.A. | 2432 | vw |  |  |  |
| N.A. | 2419 | vw |  |  |  |
| 𝜈CC and 𝛿_ip_CCH + 𝛿_ip_CCC ^**^ | 2286 | w |  |  |  |
| 𝛿_ip_CCH + 𝛿_ip_CCH ^**^ | 2268 | w |  |  |  |
| 𝛿_ip_CCH + 𝛿_ip_CCH ^**^ | 2257 | w |  |  |  |
| 𝜈CC and 𝛿_ip_CCH + 𝛿_ip_CCC ^**^ | 2234 | w |  |  |  |
| N.A. | 2199 | vw |  |  |  |
| 𝜈CC and 𝛿_ip_CCH + 𝛿_oop_CC ^**^ | 2180 | w |  |  |  |
| N.A. | 2160 | vw |  |  |  |
| 𝜈CC and 𝛿_ip_CCH + 𝛿_oop_CC ^**^ | 2147 | w |  |  |  |
| 𝜈CC and 𝛿_ip_CCH + 𝛿_oop_CC ^**^ | 2135 | w |  |  |  |
| 𝜈CC and 𝛿_ip_CCH + 𝛿_ip_CCC and 𝛿_ip_CCH ^**^ | 2102 | w |  |  |  |
| N.A. | 2089 | vw |  |  |  |
| N.A. | 2079 | vw |  |  |  |
| N.A. | 2070 | vw |  |  |  |
| 𝜈CC and 𝛿_ip_CCH + 𝛿_ip_CCC and 𝛿_ip_CCH ^**^ | 2048 | w |  |  |  |
| N.A. | 2036 | vw |  |  |  |
| N.A. | 2012 | vw |  |  |  |
| 𝛿_ip_CCH + 𝛿_ip_CCC ^**^ | 1996 | vw |  |  |  |
| N.A. | 1973 | vw |  |  |  |
| N.A. |  |  | 1960 |  |  |
| Overtone 𝛿_oop_CH ^**^ | 1944 | m | 1944 | w |  |
| 𝛿_oop_CH + 𝛿_oop_CH ^**^ | 1917 | s | 1918 | w |  |
| 𝛿_oop_CH + 𝛿_oop_CH ^**^ | 1898 | m | 1898 | w |  |
| Overtone 𝛿_oop_CH ^**^ | 1888 | m |  |  |  |
| 𝛿_oop_CH + 𝛿_oop_CH ^**^ | 1848 | m |  |  |  |
| 𝛿_oop_CH + 𝛿_oop_CH and 𝜏CC ^**^ | 1821 | m |  |  |  |
| N.A. | 1807 | m |  |  |  |
| 𝛿_oop_CH + 𝛿_oop_CH and 𝜏CC ^**^ | 1780 | s |  |  |  |
| N.A. | 1769 | s |  |  |  |
| 𝛿_oop_CH + 𝛿_oop_CH ^**^ | 1755 | s |  |  |  |
| 𝛿_ip_CCH + 𝛿_ip_CCC and 𝛿_ip_CCH ^**^ | 1620 | s |  |  |  |
| 𝜈CC and 𝛿_ip_CCH ^*/**^ | 1597 | s |  |  |  |
| 𝜈CC and 𝛿_ip_CCH ^*/**^ | 1560 | s | 1561 | w |  |
| Overtone (𝛿_ip_CCH and 𝛿_ip_CCC) ^*/**^ | 1512 | s | 1514 | w |  |
| 𝜈CC and 𝛿_ip_CCH ^*/**^ | 1493 | s | 1493 | w |  |
| 𝜈CC and 𝛿_ip_CCH ^**^ | 1474 | s | 1474 | w |  |
| 𝛿_oop_CH + 𝜏CC and 𝛿_oop_CH ^**^ | 1458 |  | 1456 | w |  |
| 𝛿_oop_CH + 𝜏CC and 𝛿_oop_CH ^*/**^ | 1427 | m |  |  |  |
| 𝜈CC and 𝛿_ip_CCH + 𝛿_ip_CCC ^*/**^ | 1416 | s | 1418 | w |  |
| 𝛿_ip_CCH ^**^ | 1410 | s | 1408 | w |  |
| 𝜈CC ^*/**^ | 1387 | m |  |  |  |
| 𝛿_ip_CCC and 𝛿_ip_CCH + 𝛿_ip_CCC and 𝛿_ip_CCH ^**^ | 1365 | m |  |  |  |
| 𝜈CC and 𝛿_ip_CCH ^**^ | 1346 | s |  |  |  |
| 𝜈CC and 𝛿_ip_CCH ^*/**^ | 1312 | s |  |  |  |
| 𝛿_ip_CCH and 𝜈CC ^**^ | 1271 | s |  |  |  |
| 𝛿_ip_CCH and 𝜈CC ^**^ | 1245 | s |  |  |  |
| 𝛿_ip_CCH and 𝜈CC ^**^ | 1236 | m |  |  |  |
| 𝛿_ip_CCH and 𝛿_ip_CCC + 𝛿_ip_CCH and 𝛿_ip_CCC ^**^ | 1213 | m |  |  |  |
| 𝛿_ip_CCH ^*/**^ | 1194 | s |  |  |  |
| 𝛿_oop_CH + 𝜏CC and 𝛿_oop_CH ^**^ | 1184 | s |  |  |  |
| 𝛿_oop_CH + 𝜏CC and 𝛿_oop_CH ^**^ | 1179 | s |  |  |  |
| 𝛿_ip_CCH ^*/**^ | 1163 | m |  |  |  |
| 𝛿_ip_CCH ^*/**^ | 1148 | m |  |  |  |
| 𝛿_oop_CH and 𝜏CC + 𝛿_oop_CH and 𝜏CC ^**^ | 1121 | m |  |  |  |
| 𝛿_ip_CH + 𝜈CC ^*^ | 1111 | m |  |  |  |
| 𝛿_oop_CH and 𝜏CC + 𝛿_oop_CH and 𝜏CC ^**^ | 1081 | m |  |  |  |
| N.A. | 1071 | m |  |  |  |
| N.A. | 1053 | vw |  |  |  |
| 𝛿_ip_CCC + 𝛿_ip_CCC and 𝛿_ip_CCH ^*/**^ | 1035 | m |  |  |  |
| 𝜈CC and 𝛿_ip_CCH ^*/**^ | 1022 | m |  |  |  |
| N.A. | 997 | vw |  |  |  |
| 𝜈CC and 𝛿_ip_CCH ^**^ | 984 | m |  |  |  |
| 𝛿_oop_CH ^*/**^ | 974 | m |  |  |  |
| 𝛿_oop_CH ^**^ | 962 | m |  |  |  |
| 𝛿_oop_CH ^**^ | 945 | m |  |  |  |
| N.A. | 924 | w |  |  |  |
| 𝛿_ip_CCC and 𝛿_ip_CCH ^*/**^ | 889 | m | 887 | w |  |
| 𝛿_oop_CH and 𝜏CC ^*/**^ | 876 | m | 880 | w |  |
| 𝛿_oop_CH and 𝜏CC ^*/**^ | 847 | m |  |  |  |
| 𝛿_oop_CH and 𝜏CC ^**^ | 836 | m |  |  |  |
| 𝛿_ip_CCH and 𝛿_ip_CCC ^**^ | 821 | m | 826 | w | +5 |
| 𝛿_oop_CH ^**^ | 814 | m |  |  |  |
| 𝜏CC and 𝛿_oop_CH ^**^ | 793 | m |  |  |  |
| 𝛿_oop_CH ^**^ | 764 | m |  |  |  |
| 𝛿_oop_CH ^*/**^ | 742 | m |  |  |  |
| N.A. | 699 | m |  |  |  |
| 𝜏CC and 𝛿_oop_CH ^*/**^ | 689 | m |  |  |  |
| 𝜏CC and 𝛿_oop_CH ^*/**^ | 670 | m |  |  |  |
| 𝛿_ip_CCC and 𝛿_ip_CCH ^*/**^ | 637 | m |  |  |  |
| N.A. | 614 | w |  |  |  |
| N.A. | 583 | vw |  |  |  |
| 𝛿_ip_CCC ^**^ | 561 | m |  |  |  |
| 𝜏CC and 𝛿_oop_CH ^*/**^ | 534 | m |  |  |  |
| 𝛿_ip_CCC ^**^ | 513 | m |  |  |  |
| N.A. | 499 | m |  |  |  |
| 𝛿_ip_CCC and 𝛿_ip_CCH ^**^ | 479 | m |  |  |  |
| 𝜏CC and 𝛿_oop_CH ^*/**^ | 455 | m |  |  |  |

* ^2^

** DFT calculation, this work

N.A. = Not Available


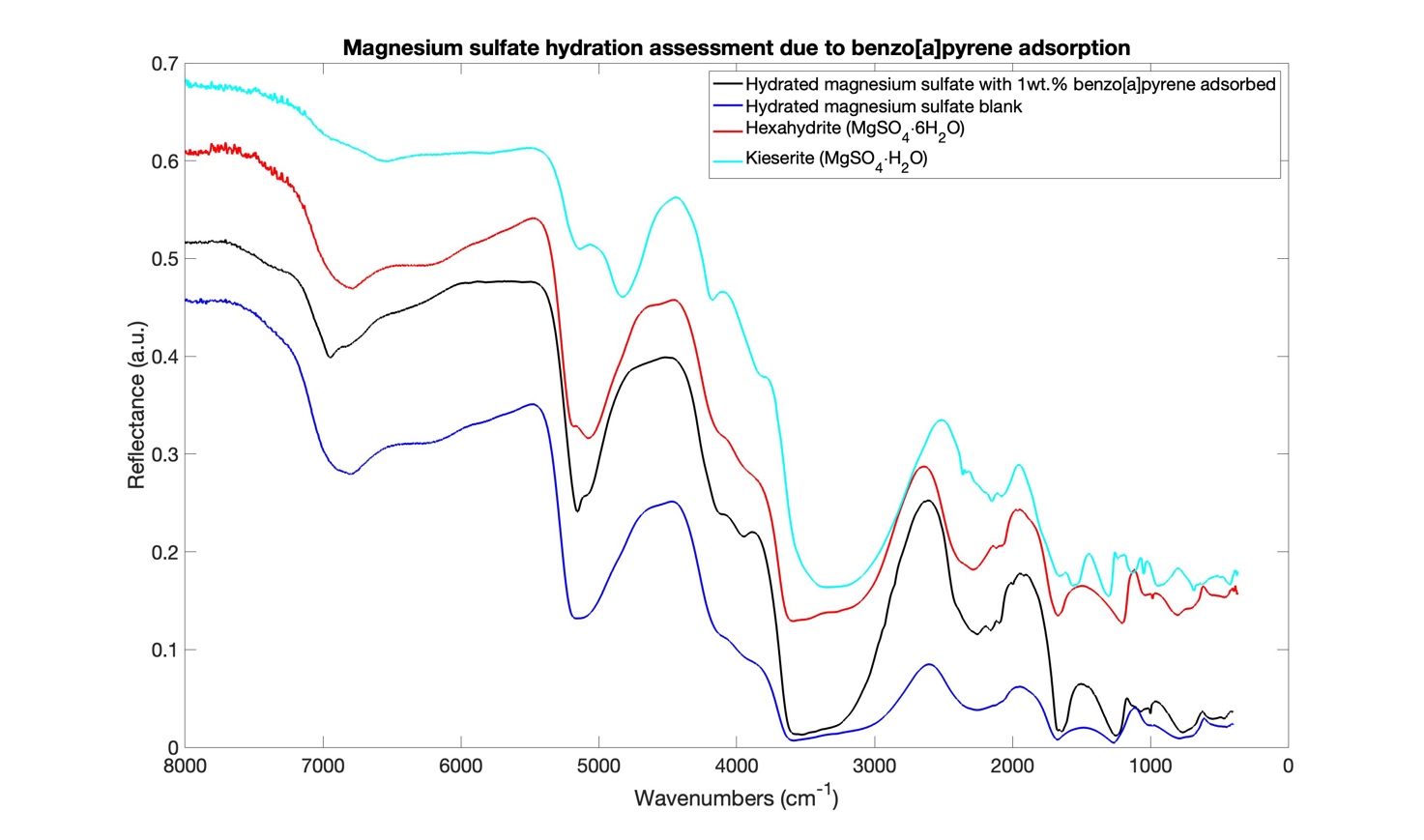


1st overtone of the

water OH stretching

Combination of water

OH stretching and OH bending

OH stretching band

**Figure S2**: IR spectra of hydrated magnesium sulfate with 1wt.% benzo[a]pyrene, hydrated magnesium sulfate blank, hexahydrite (MgSO4∙6H2O) and kieserite (MgSO4∙H2O) in order to assess the hydration state of the first two samples and investigate the organic role in the spectral changes.

**Table S3 – Weighting procedure for obtaining the weighted average irradiation values (at the bottom of Table 1 and Table 2) where** $\mathbf{Err}_{\boldsymbol{i}}$ **and** $\boldsymbol{Val}_{\boldsymbol{i}}$ **are the error and the value of each irradiation parameter, respectively.**

| Weight ($\mathbf{W}_{\boldsymbol{i}}$) | $\mathbf{W}_{\boldsymbol{i}}\mathbf{=}\frac{\mathbf{1}}{\mathbf{(}{\mathbf{Err}_{\boldsymbol{i}}}^{\mathbf{2}}\mathbf{)}}$ |
| --- | --- |
| Mean value ($\overline{\boldsymbol{Val}}$) | $\overline{\boldsymbol{Val}}=\frac{\sum_{i}^{n} (W_{i}\cdot{Val}_{i})}{\sum_{i}^{n} (W_{i})}$ |
| Mean error ($\overline{\boldsymbol{Err}}$) | $\overline{Err}=\sqrt{\frac{1}{\sum_{i}^{n} W_{i}}}$ |


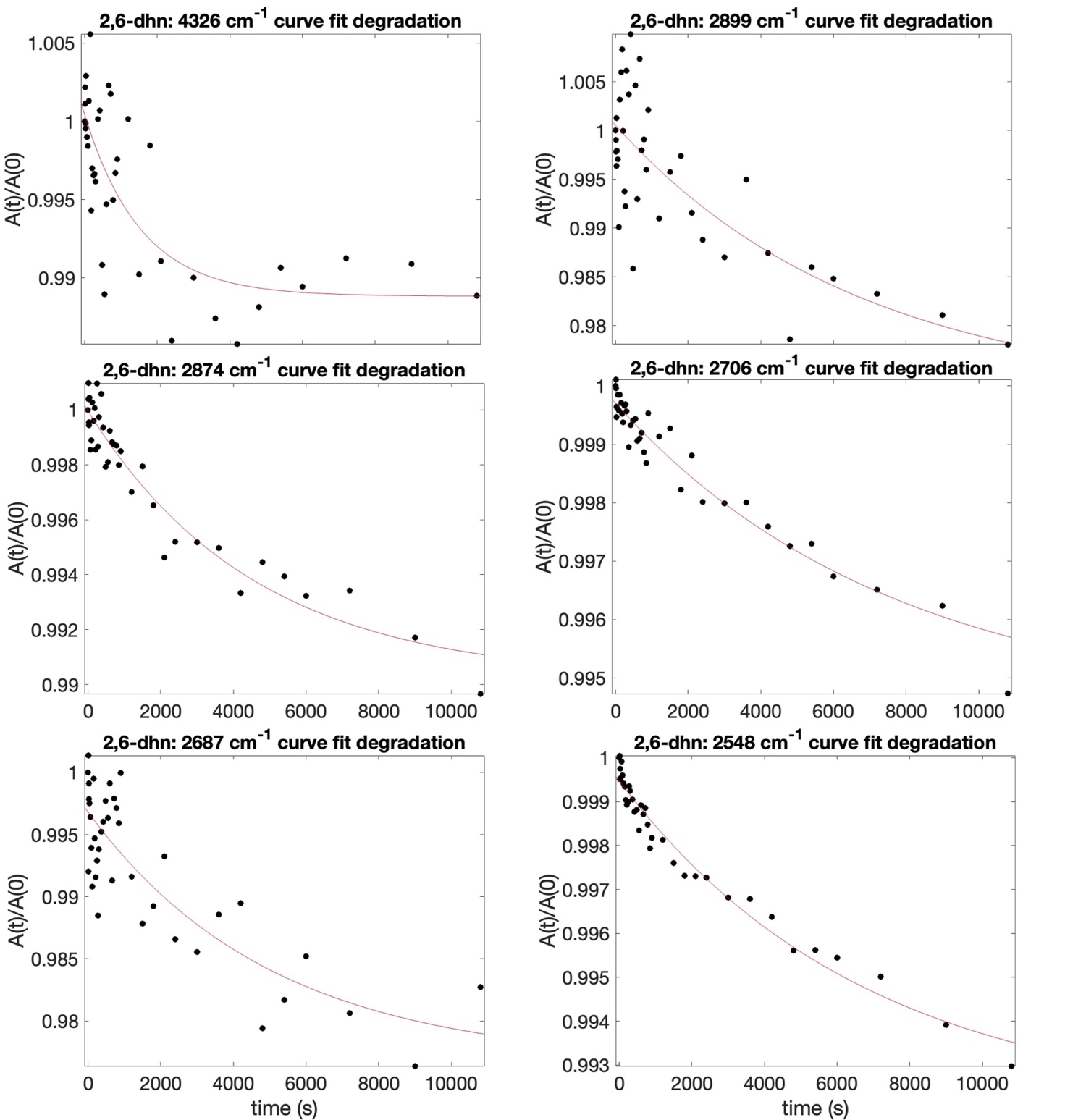


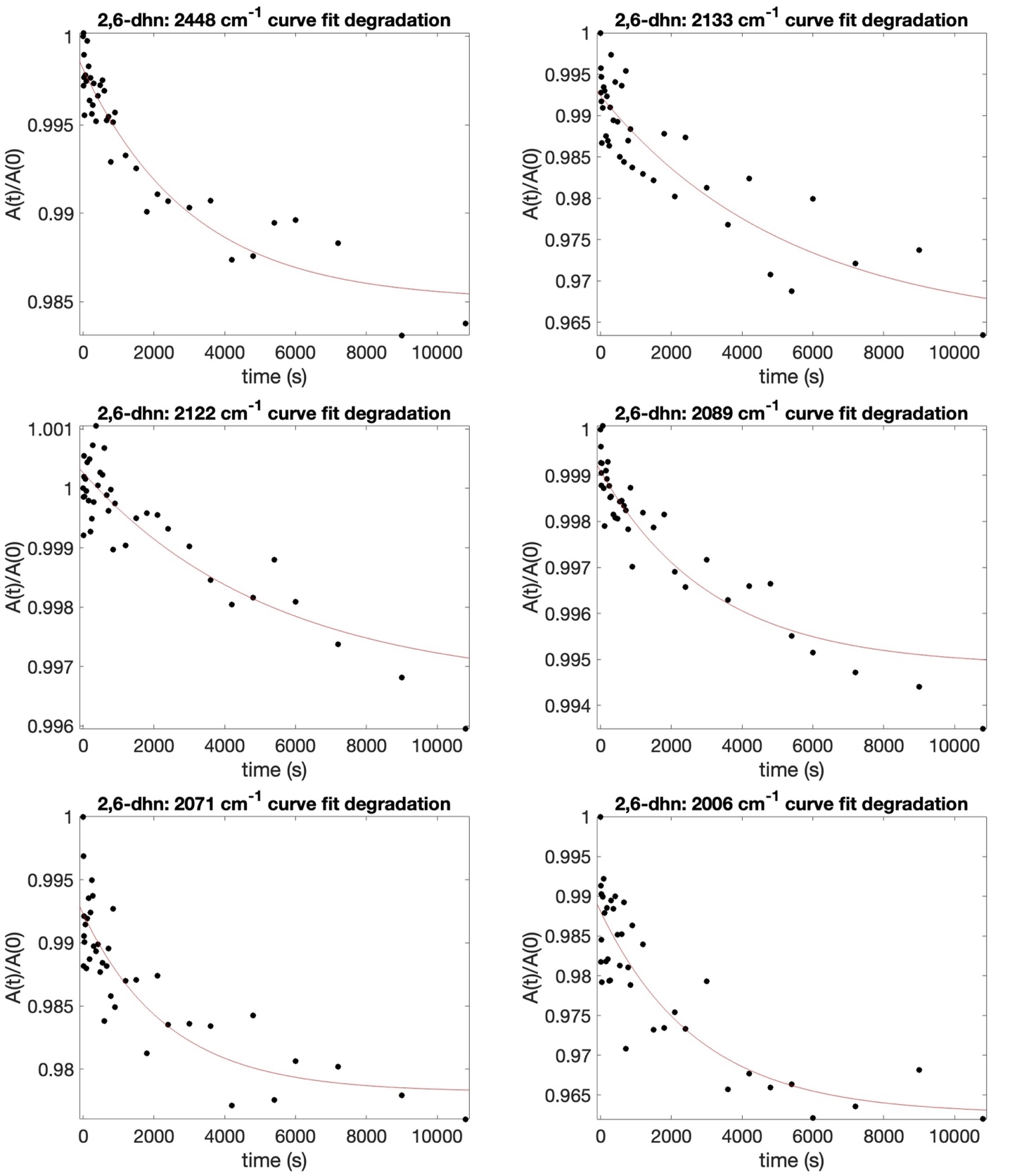


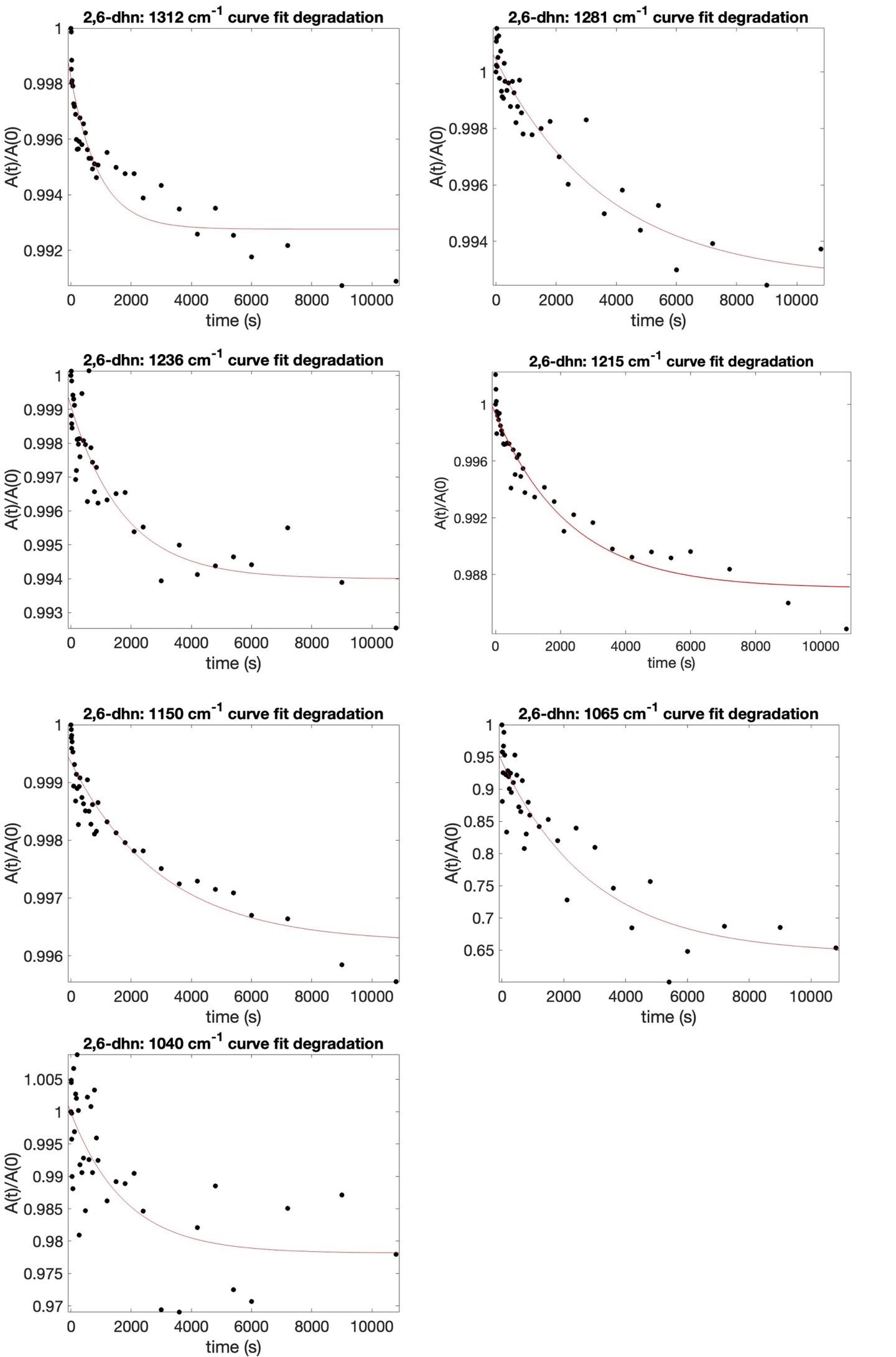


**Figure S3**: Curve fit degradation results for pure 2,6-dihydroxynaphthalene molecule during UV irradiation experiment


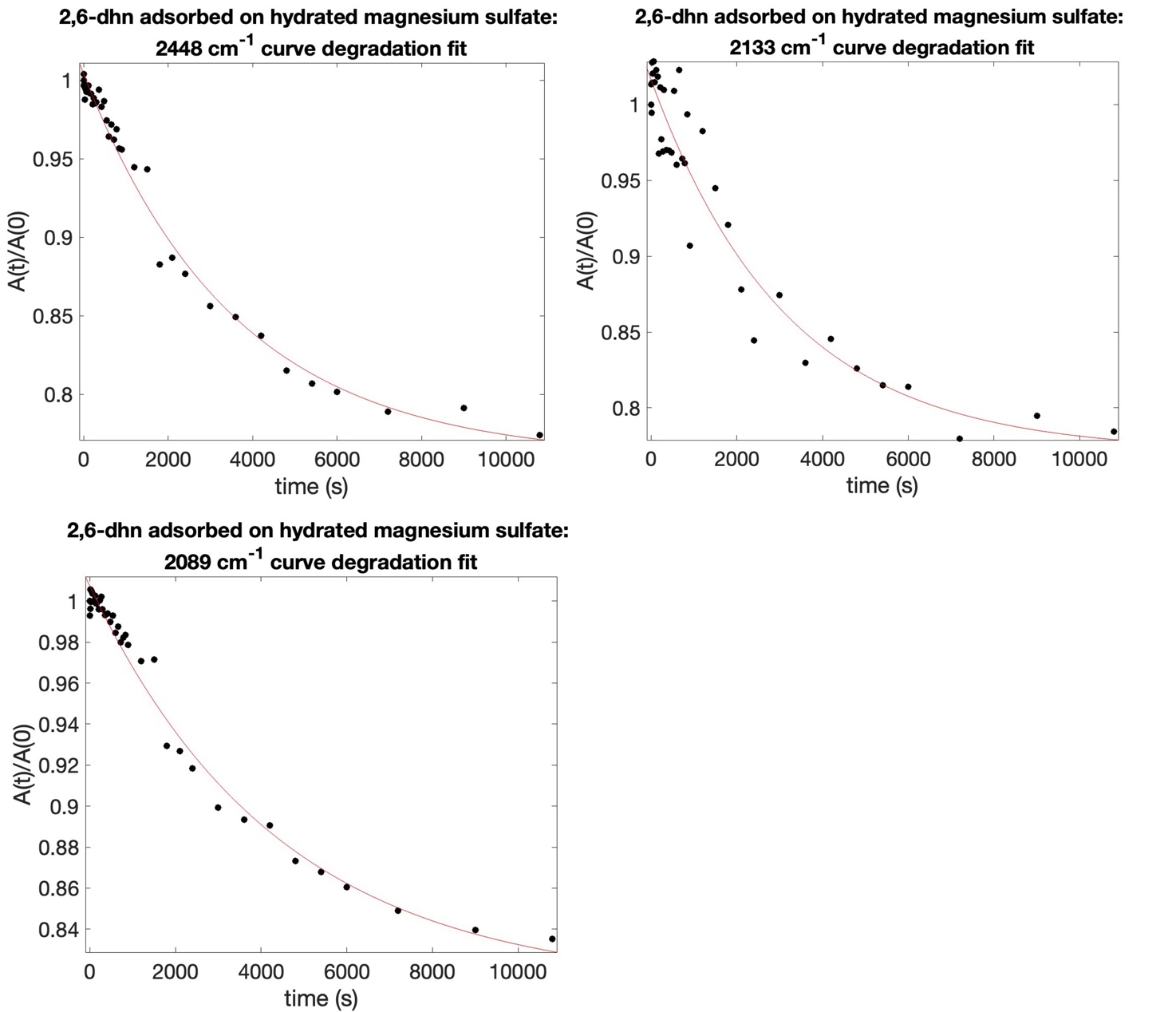


**Figure S4**: Curve fit degradation results for 10 wt.% 2,6-dihydroxynaphthalene adsorbed on hydrated magnesium sulfate during UV irradiation experiment


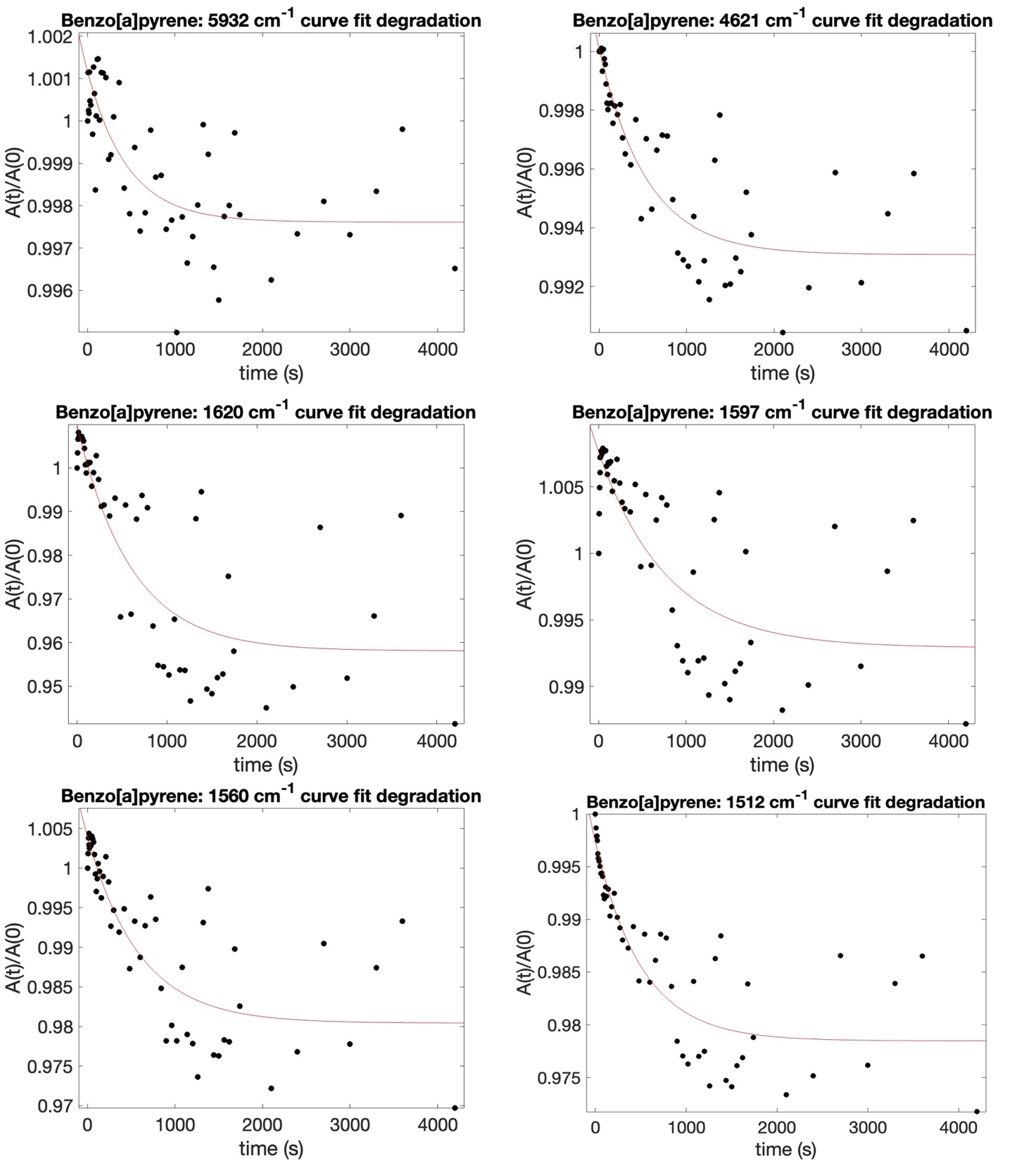


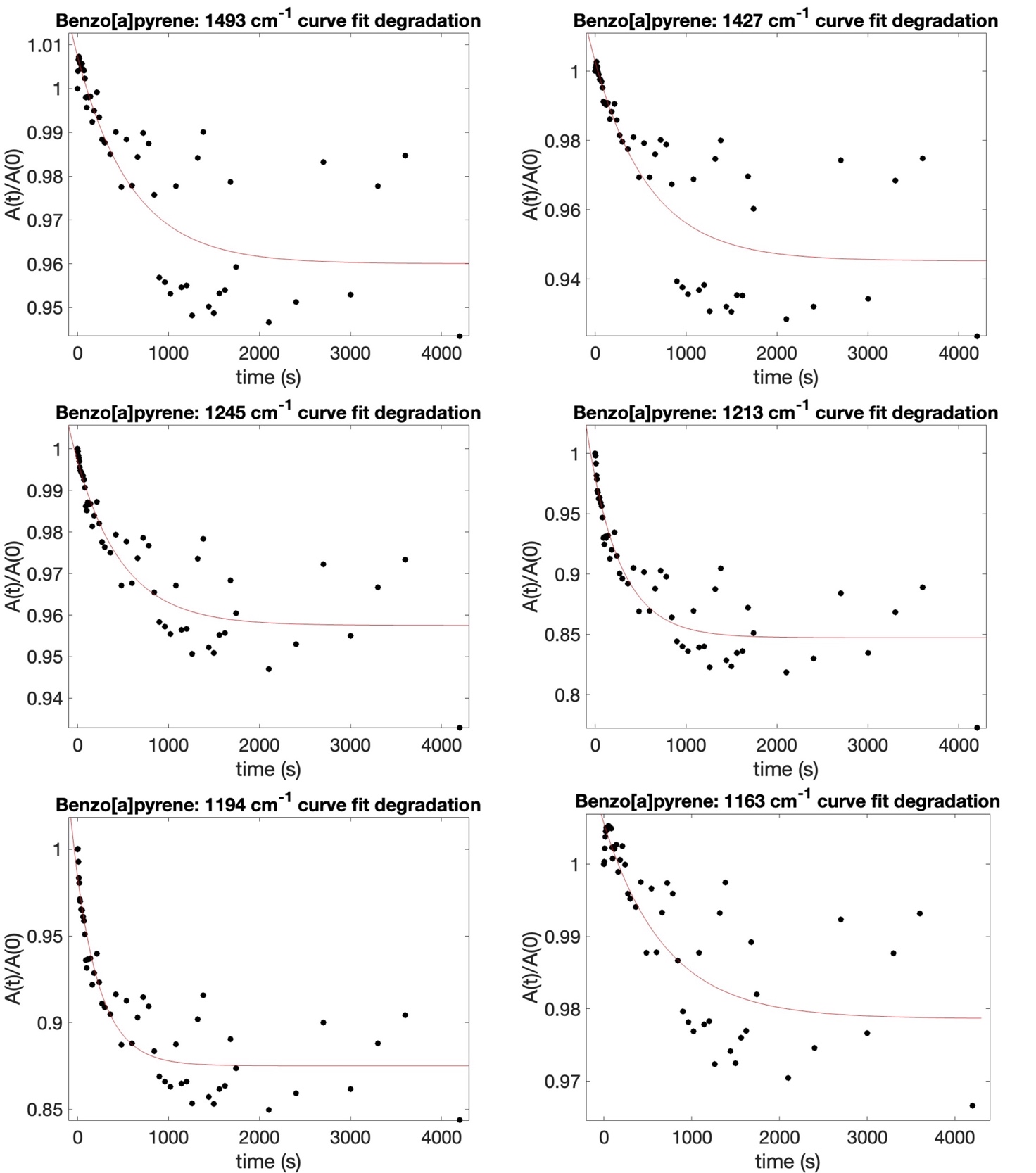


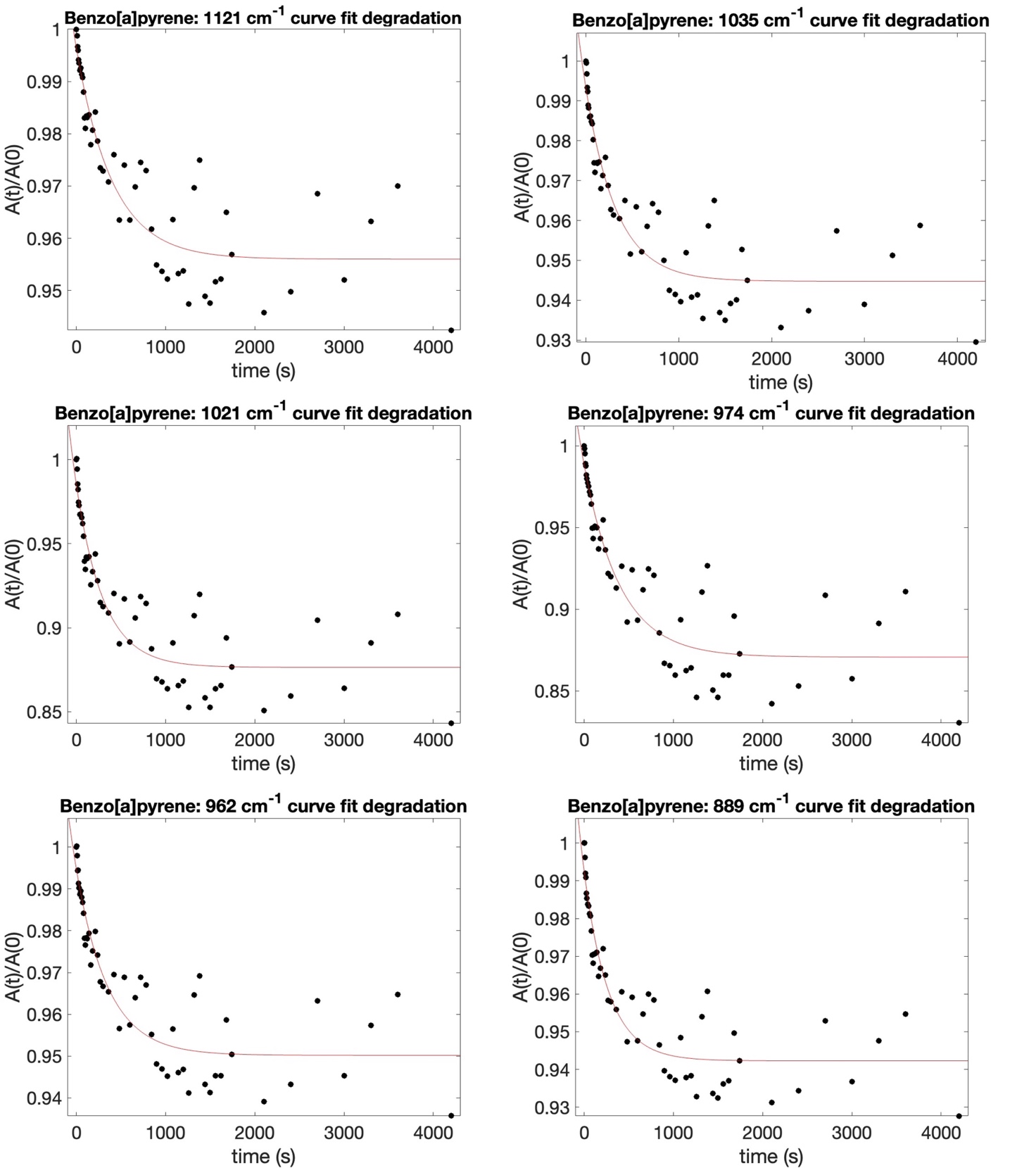


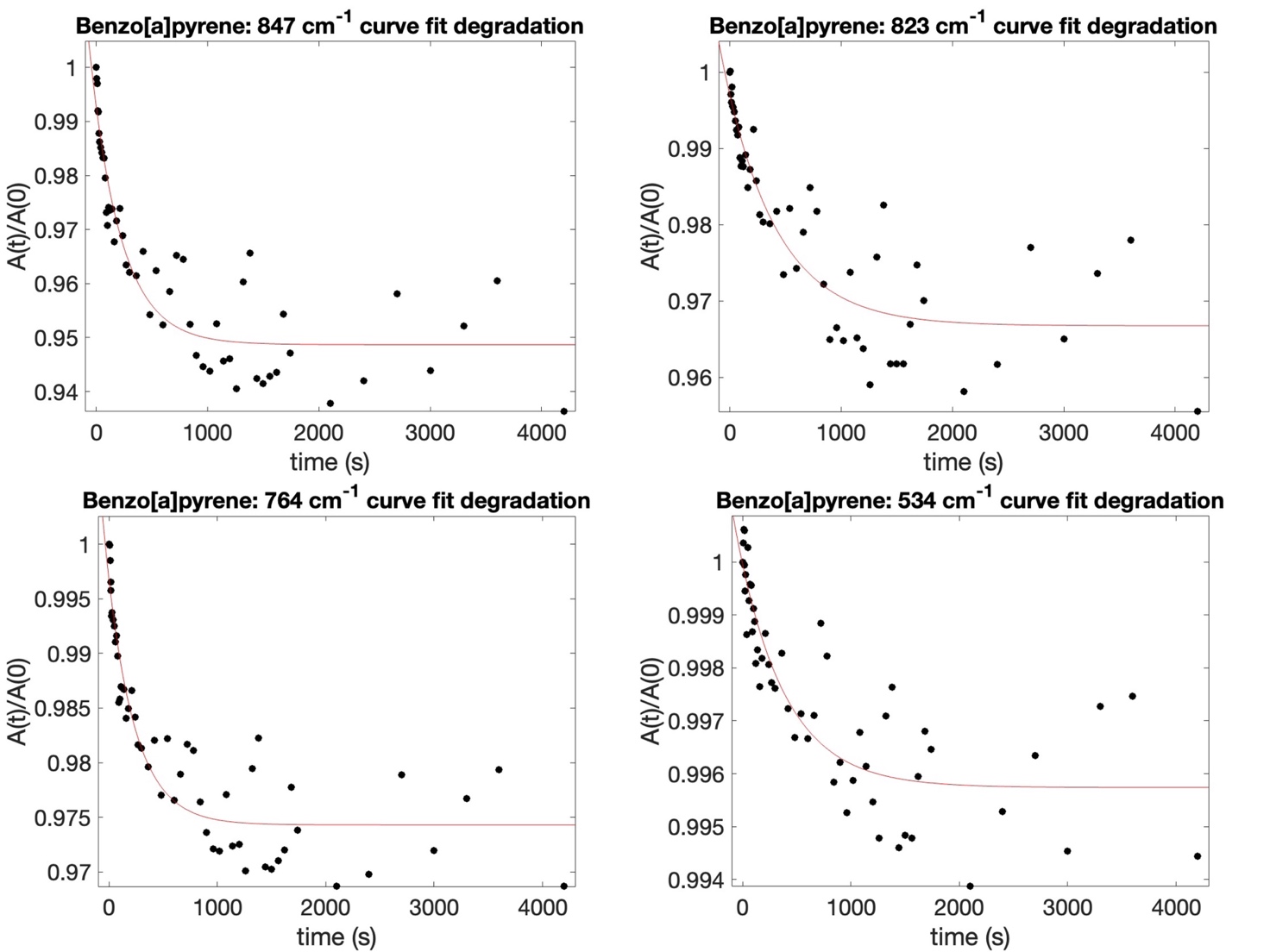


**Figure S5**: Curve fit degradation results for pure benzo[a]pyrene molecule during UV irradiation experiment

**Table S4 – Bands of 2,6-dihydroxynaphthalene and benzo[a]pyrene when adsorbed on hydrated magnesium sulfate in the IR SuperCam spectral range (**$\boldsymbol{7700-3850}$ $\boldsymbol{cm}^{\boldsymbol{-1}}$**,** $\boldsymbol{1.3-2.6}$ $\boldsymbol{\mu m}$**) with the vibrational mode assignment and intensity (w = weak; m = medium).**

| 2,6-dihydroxynaphthalene vibrational mode | Wavenumber [cm^-1^] | Wavelength [$\boldsymbol{\mu m}$] | Intensity |
| --- | --- | --- | --- |
| 𝜈CH + 𝜈CH** | 5960 | 1.68 | s |
| 𝛿_ip_CH and 𝛿_ip_OH + 𝜈OH** | 4812 | 2.08 | w |
| 𝛿_ip_CH + 𝜈OH** | 4765 | 2.10 | w |
| 𝜈CH and 𝛿_ip_OH and 𝛿_ip_CH + 𝜈CH** | 4682 | 2.14 | w |
| 𝜈CH and 𝛿_ip_OH and 𝛿_ip_CH + 𝜈CH** | 4657 | 2.15 | s |
| 𝜈CH and 𝛿_ip_OH + 𝜈CH** | 4636 | 2.16 | w |
| 𝛿_ip_CH and 𝜈CH and 𝛿_ip_OH + 𝜈CH** | 4581 | 2.18 | w |
| 𝛿_ip_CH and 𝜈CH and 𝜈OH + 𝜈CH** | 4560 | 2.19 | w |
| 𝛿_oop_CH + 𝜈OH** | 4536 | 2.20 | w |
| 𝛿_oop_CH + 𝜈OH** | 4513 | 2.21 | w |
| 𝛿_ip_CH and 𝛿_ip_OH + 𝜈CH** | 4428 | 2.26 | m |
| 𝛿_ip_CH and 𝛿_ip_OH + 𝜈CH * | 4328 | 2.31 | m |
| 𝜈C-OH and ring breathing + 𝜈CH * | 4309 | 2.32 | s |
| 𝛿_ip_CH and 𝛿_ip_OH + 𝜈CH** | 4216 | 2.37 | m |
| 𝛿_ip_CH + 𝜈CH * | 4189 | 2.39 | m |
| 𝛿_ip_CH and 𝜈CH + 𝜈CH** | 4031 | 2.48 | w |
| 𝛿_ip_C-OH + 𝜈OH** | 4010 | 2.49 | m |
| 𝛿_ip_CH and 𝜈CH + 𝜈CH** | 3987 | 2.51 | w |
| 𝛿_oop_OH + 𝜈OH** | 3977 | 2.51 | w |

* ^1^

** DFT calculations (this work)

| Benzo[a]pyrene vibrational mode | Wavenumber [cm^-1^] | | Wavelength [$\boldsymbol{\mu m}$] | | Intensity | |  |
| --- | --- | --- | --- | --- | --- | --- | --- |
| Overtone 𝜈CH ^**^ | | 5939 | | 1.68 | | s | |
| 𝜈CH + 𝜈CC and 𝛿_ip_CCH ^**^ | | 4621 | | 2.16 | | w | |
| 𝜈CH + 𝜈CC and 𝛿_ip_CCH ^*/**^ | | 4492 | | 2.23 | | s | |

* ^2^

** DFT calculation (this work)

N.A. = Not Available

**Supplementary Note “UV lamp details”**

Regarding the concern about potential sample heating due to the infrared tail of the spectrum, we have to consider that the effective output power of the lamp, excluding the fiber optics, is on the order of watts. However, the presence of the optical fiber reduces the output power by two orders of magnitude. Specifically, in the 1000–2200 nm range, the spectral power density is approximately 0.03 mW/nm, resulting in an integrated power of about 36 mW.


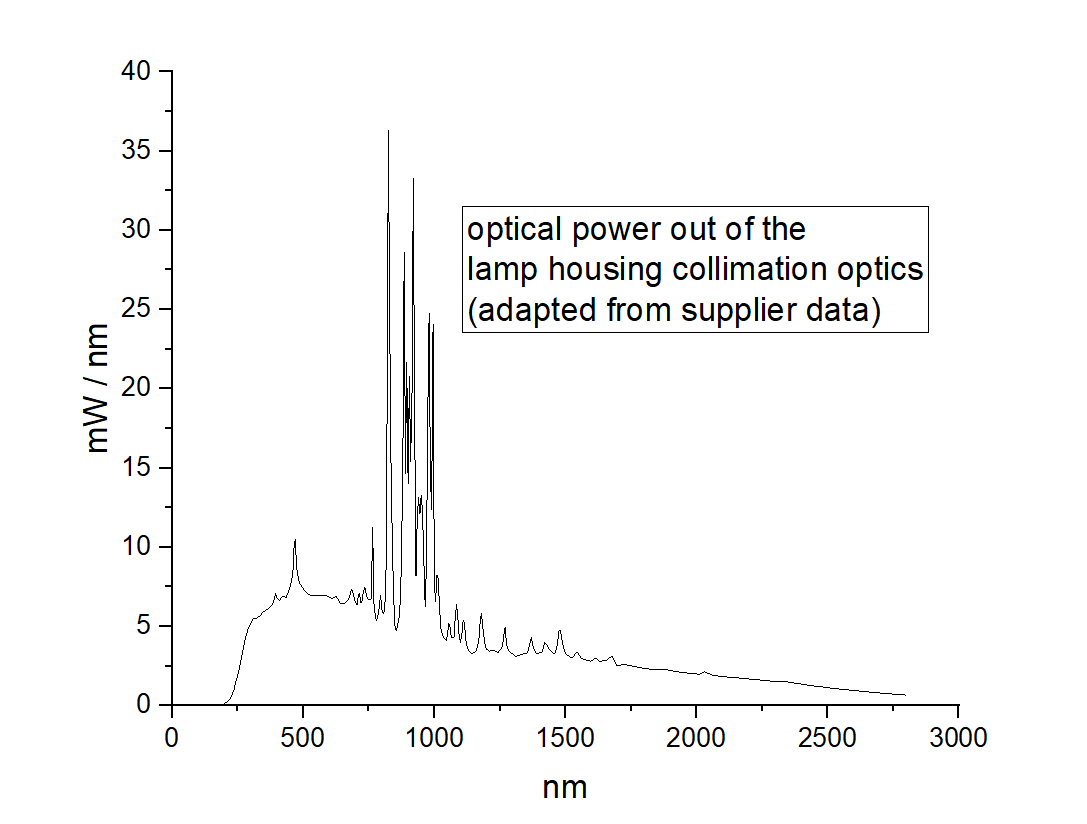


**Figure S6**: the radiance of the Newport Oriel 300W Xenon discharge lamp.

Moreover, the radiation that does reach the sample within this spectral range is possibly absorbed only by vibrational overtones characterized by low absorption cross sections. Therefore, the radiation in the 1000-2200 nm spectral range is poorly absorbed by the sample.

Furthermore, the radiation beyond 2200 nm is poorly transmitted by the optical fiber due to the presence of OH groups in the fiber structure~~.~~ Thus, we are confident that sample heating due to the infrared emission of the Xe lamp is not a relevant issue in our experimental setup.


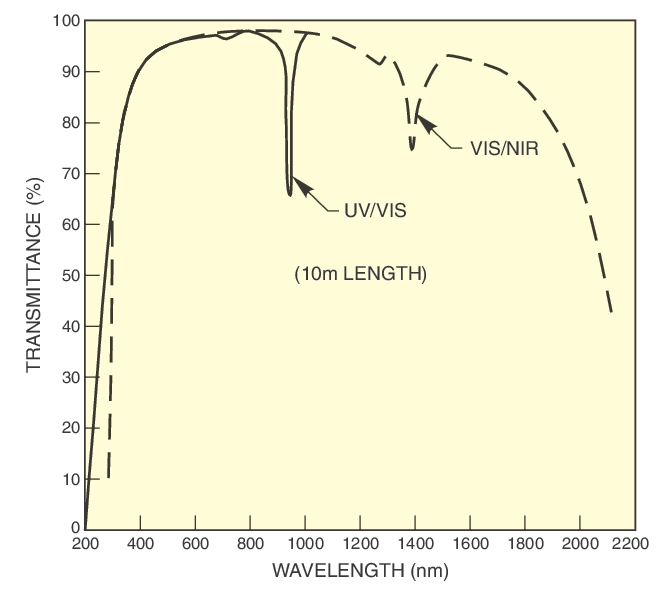


**Figure S7**: Transmittance data for UV-vis and vis-NIR large core fibers.

In order to further clarify this aspect, a test was performed in our laboratory to evaluate potential heating effects. Specifically, a thermocouple was inserted into powder micrometer size of hydrated magnesium sulfate sample placed inside the sample holder. The same experimental conditions were then reproduced, irradiating the sample for the same amount of time of the experiments described in the text.

Under these conditions, the initial temperature was recorded with the lamp switched off. Subsequently, after turning on the lamp, temperatures were monitored at increasing time intervals, following the same irradiation time steps used in the manuscript experiments. The resulting temperature profiles are shown below for irradiation durations of 5 seconds, 10 seconds, 20 seconds, 30 seconds, 1 minute, 5 minutes, 10 minutes, 20 minutes and 30 minutes.

The results indicate a maximum temperature increase of 1.3°C. Based on this outcome, we considered such temperature increase negligible in affecting the photostability of 2,6-DHN and benzo[a]pyrene analyzed in this study.


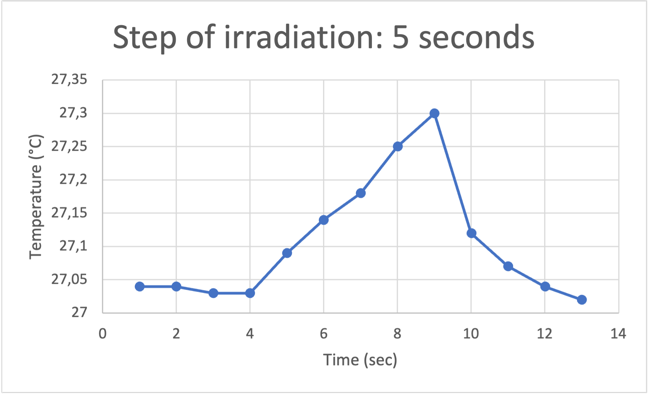

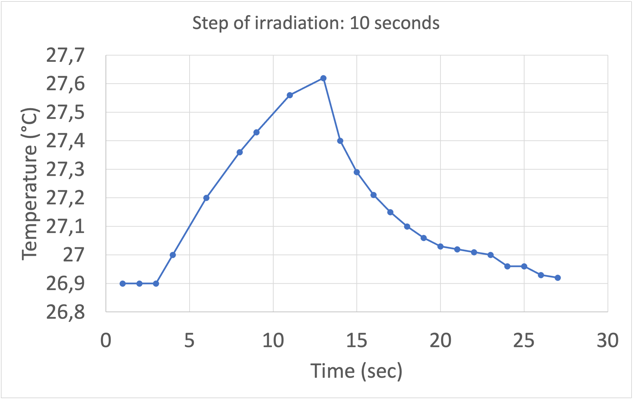


Lamp

off

Lamp off

Lamp on

Lamp on

Lamp off

Lamp off


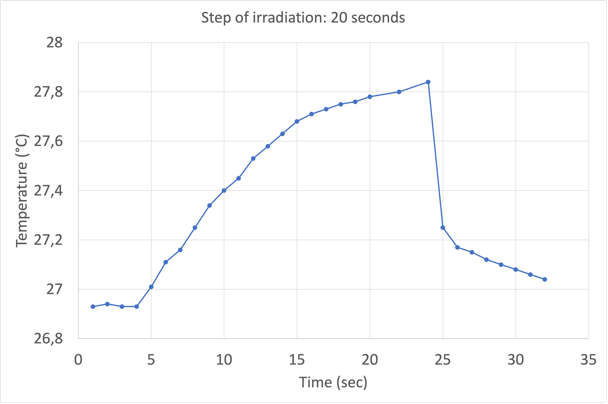

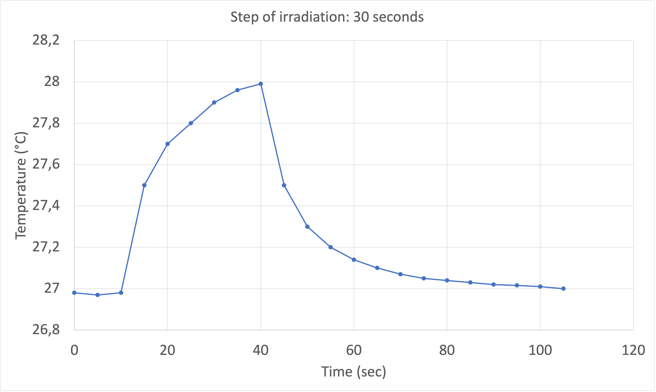


Lamp on

Lamp

off

Lamp

off

Lamp off

Lamp off

Lamp on

Lamp

off


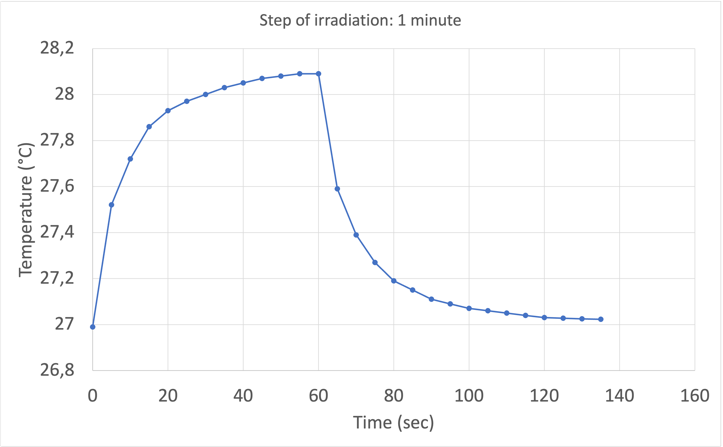

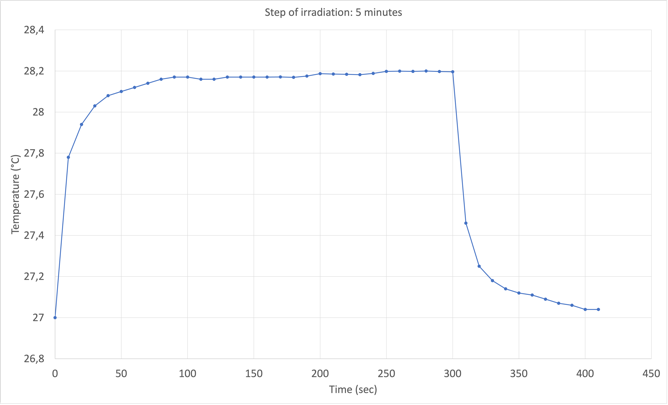


Lamp off

Lamp on

Lamp

off

Lamp off

Lamp on


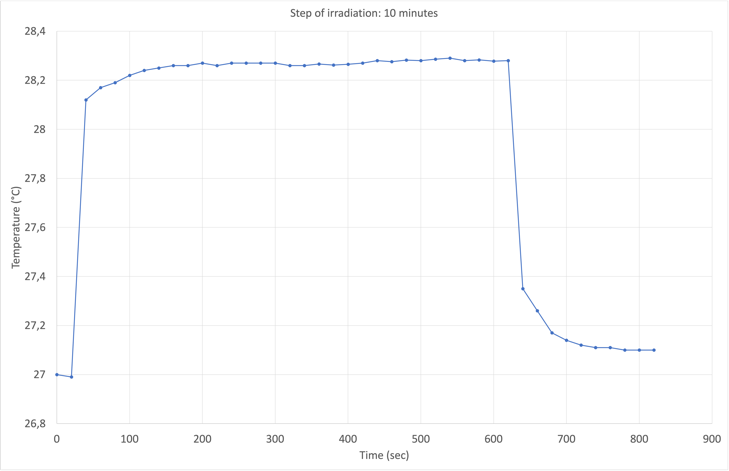

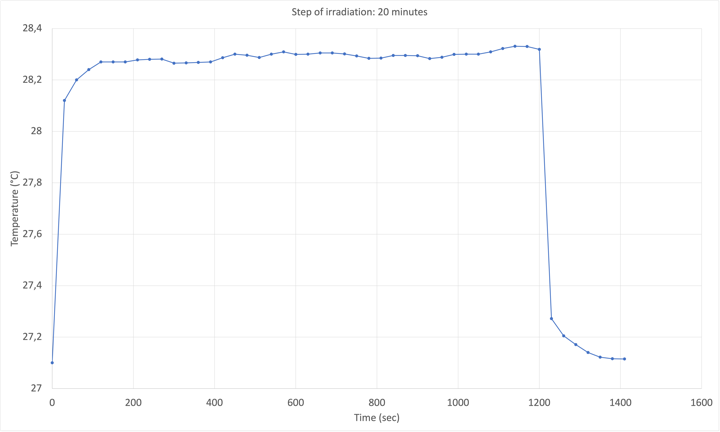


Lamp

off

Lamp off

Lamp

off

Lamp on

Lamp off

Lamp on


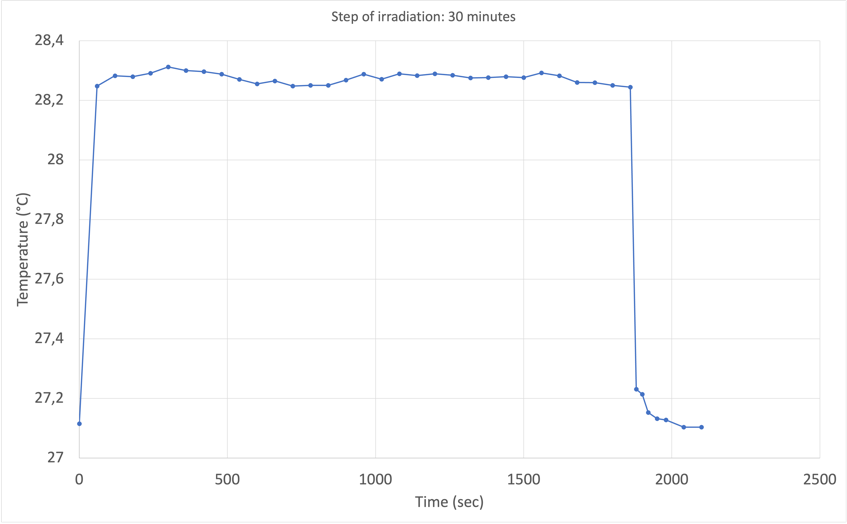


Lamp off

Lamp on

Lamp

off

**Figure S8**: the resulting temperature profiles for irradiation durations of 5 seconds, 10 seconds, 20 seconds, 30 seconds, 1 minute, 5 minutes, 10 minutes, 20 minutes and 30 minutes.

**
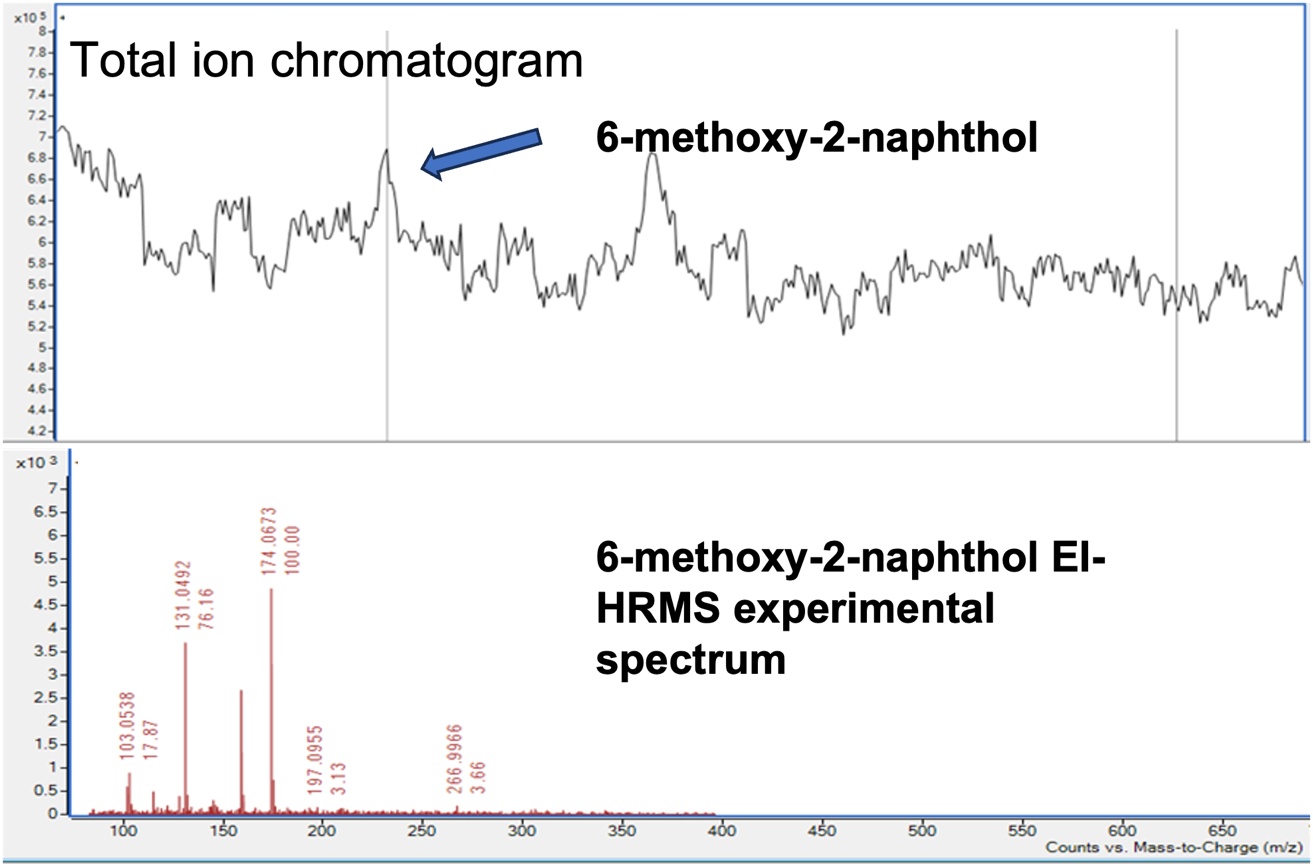
**

**
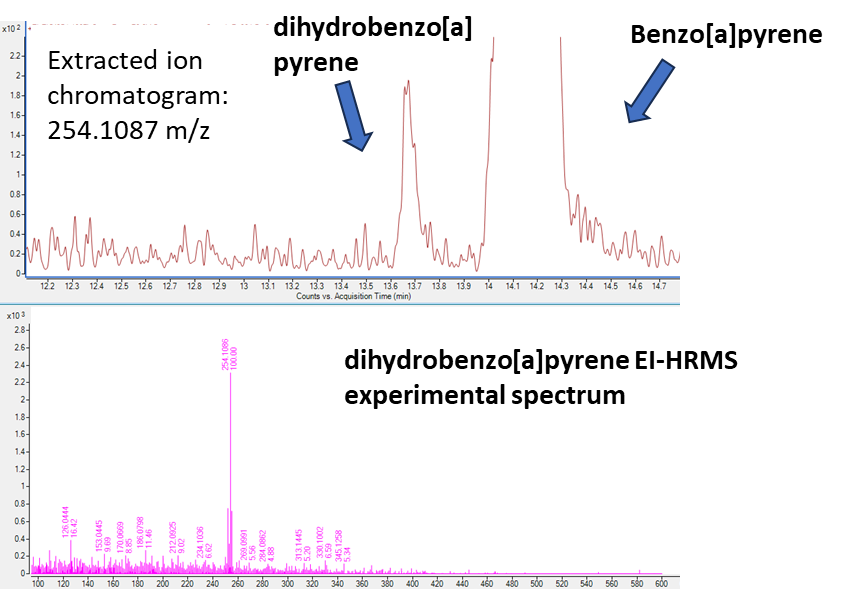
**

**Figure S9**. Up: 10wt.% 2,6-dihydroxynaphthalene adsorbed on hydrated magnesium sulfate after the UV irradiation. Extraction of ion chromatogram of 6-methoxy-2-naphthol is shown;

Down: 1wt.% benzo[a]pyrene adsorbed on hydrated magnesium sulfate after 4200 seconds of UV irradiation. Extraction of ion chromatogram of dihydrobenzo[a]pyrene and benzo[a]pyrene is shown.

**
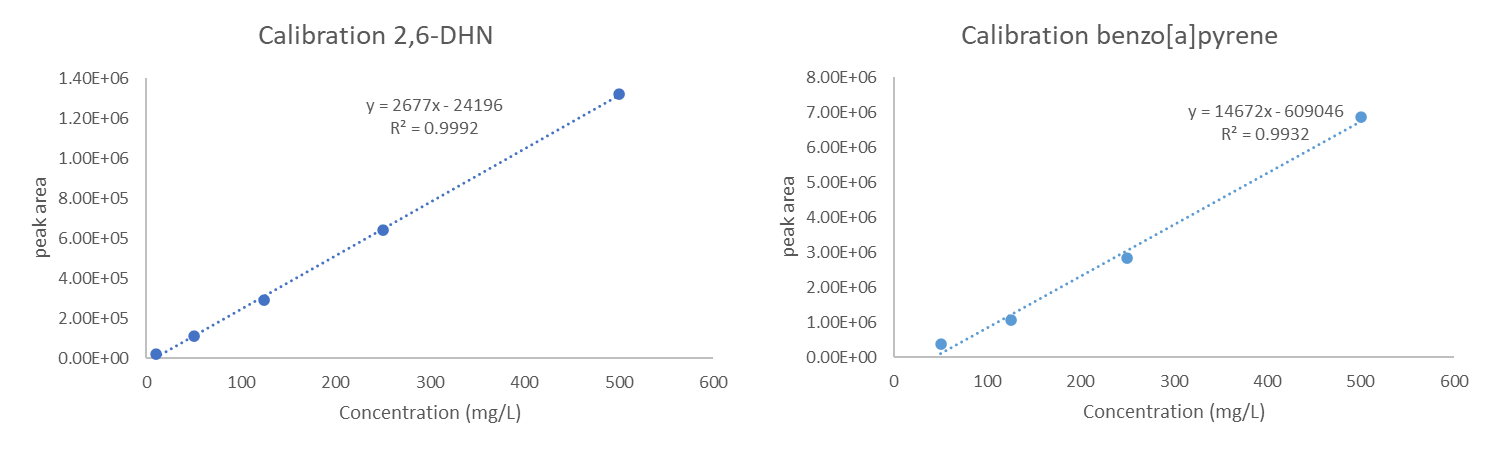
**

**Figure S10**: calibration of 2,6-dhn and benzo[a]pyrene with LC-LEI-HRMS.

1. Sharma, O. P. + Singh, R. D. Vibrational Spectra and Assignments of 1 Hydroxy, 2 Hydroxy and 2, 6 Dihydroxy Naphthols. *Indian Journal of Physics* **51**, 93–98 (1977).

2. Onchoke, K. K., Hadad, C. M. + Dutta, P. K. Structure and vibrational spectra of mononitrated benzo [a] pyrenes. *J Phys Chem A* **110**, 76–84 (2006).
